# Multiple Roles and Interactions of *Tbx4* and *Tbx5* in Development of the Respiratory System

**DOI:** 10.1371/journal.pgen.1002866

**Published:** 2012-08-02

**Authors:** Ripla Arora, Ross J. Metzger, Virginia E. Papaioannou

**Affiliations:** 1 Department of Genetics and Development, Columbia University Medical Center, New York, New York, United States of America; 2 Department of Anatomy, School of Medicine, University of California San Francisco, San Francisco, California, United States of America; Stanford University School of Medicine, United States of America

## Abstract

Normal development of the respiratory system is essential for survival and is regulated by multiple genes and signaling pathways. Both *Tbx4* and *Tbx5* are expressed throughout the mesenchyme of the developing lung and trachea; and, although multiple genes are known to be required in the epithelium, only Fgfs have been well studied in the mesenchyme. In this study, we investigated the roles of *Tbx4* and *Tbx5* in lung and trachea development using conditional mutant alleles and two different Cre recombinase transgenic lines. Loss of *Tbx5* leads to a unilateral loss of lung bud specification and absence of tracheal specification in organ culture. Mutants deficient in *Tbx4* and *Tbx5* show severely reduced lung branching at mid-gestation. Concordant with this defect, the expression of mesenchymal markers *Wnt2* and *Fgf10*, as well as Fgf10 target genes *Bmp4* and *Spry2*, in the epithelium is downregulated. Lung branching undergoes arrest *ex vivo* when *Tbx4* and *Tbx5* are both completely lacking. Lung-specific *Tbx4* heterozygous;*Tbx5* conditional null mice die soon after birth due to respiratory distress. These pups have small lungs and show severe disruptions in tracheal/bronchial cartilage rings. *Sox9*, a master regulator of cartilage formation, is expressed in the trachea; but mesenchymal cells fail to condense and consequently do not develop cartilage normally at birth. *Tbx4*;*Tbx5* double heterozygous mutants show decreased lung branching and fewer tracheal cartilage rings, suggesting a genetic interaction. Finally, we show that *Tbx4* and *Tbx5* interact with *Fgf10* during the process of lung growth and branching but not during tracheal/bronchial cartilage development.

## Author Summary

Defective development of the mammalian respiratory system can lead to tracheal, bronchial, or pulmonary malformations causing severe consequences at birth or during postnatal life. Studies using mouse genetics have begun to reveal complex regulatory mechanisms that guide the development of the respiratory system, but understanding how disruption of these mechanisms leads to malformations is far from complete. In this study, we analyze the role of two T-box transcription factors, *Tbx4* and *Tbx5*, in three processes essential to the development of the respiratory system: the specification of the lung and trachea primordia, the growth and branching of the airways to form the lung, and formation of cartilage rings of the trachea and bronchi. In the absence of *Tbx5*, only one lung is specified, and no trachea. Both *Tbx4* and *Tbx5* regulate the process of lung branching by controlling the expression of the secreted growth factor *Fgf10* and activation of Fgf10 signaling. In the trachea, both *Tbx4* and *Tbx5* are important for condensation of cells to form cartilage rings, although this is regulated by a distinct pathway that does not involve Fgf10.

## Introduction

The development of the respiratory system represents an evolutionary hallmark that allowed vertebrates to survive on land utilizing air as a source of oxygen. Because the respiratory system is dispensable for embryonic survival in mammals, defects in development of the respiratory system manifest at or after birth. Indeed, abnormal development of the respiratory system in humans is associated with multiple disorders such as tracheal/bronchial atresia, tracheoesophageal fistula, bronchogenic cysts, pulmonary/lobar atresia and pulmonary hypoplasia [1]. Thus, it is important to understand the genetic basis of development of the respiratory system.

In the mouse embryo, the endodermal foregut tube is patterned by signals from the lateral plate mesoderm leading to specification of the lung and trachea at embryonic day (E) 9.0 (19–24 somites). *Nkx2.1* has been identified as the earliest marker of lung endoderm specification. At E9.25 (25–28 somites), the primary lung buds appear as ventro-lateral outpouchings of the foregut connected ventrally by the tracheal primordium. The lung buds grow in a ventro-posterior direction and continue to elongate until E11.5. The point of connection of the lung buds is thought to be the origin of the tracheal tube, which separates from the esophagus in a caudal to cranial direction by E11.5 [2], [3]. In the mouse, the left lung bud remains a single lobe and the right lung bud forms 4 lobes - cranial, medial, caudal and accessory [2]. The airways undergo a stereotypic pattern of branching beginning at E11.5 [4]; development and maturation of the alveoli occurs later.

Genes involved in different signaling pathways, including *Wnt2*, *Fgf10*, *Bmp4*, *Shh* and retinoic acid synthesis genes, have been shown to play important roles in lung specification and branch formation. Complete absence of both *Wnt2* and *Wnt2b* in mesenchyme surrounding the anterior foregut or absence of β-catenin in the foregut epithelium leads to a loss of specification of lung primordia as seen by the absence of *Nkx2.1* expression [5]–[7]. Embryos lacking *Fgf10*, which is normally expressed in mesenchyme surrounding the epithelial branching tips, form a short trachea but have no lungs [8], [9]. Inhibition of epithelial *Bmp4* signaling by overexpression of *Xnoggin* leads to a decrease in lung size and irregularly shaped lung lobes [10]. *Shh* null mutant mice have only a rudimentary lung sac due to branching severe branching defect [11]. Additionally, conditional inactivation of *Shh* in lung epithelial cells leads to the formation of hypoplastic lungs with reduced branching of the peripheral tubules [12]. Retinoic acid receptor (RAR) α and RAR β2 double null mutants show left lung agenesis and a hypoplastic right lung at E18.5 [13]. Thus, genes expressed in both the mesenchyme and the epithelium are essential for correct lung bud specification and branching.

After E11.5, mesenchyme surrounding the dorsal aspect of the trachea differentiates into the trachealis smooth muscle. Mesenchyme surrounding the ventral aspect of the trachea and lateral aspect of the main stem bronchi segments and differentiates into C shaped rings composed of chondrocytes. Ventral tracheal cartilage is formed by migration of cells that undergo mesenchymal condensation [14]. *Sox9* has been implicated as an important regulator of mesenchymal condensation and chondrocyte differentiation [15], [16]. In chondrocyte cultures it has been shown that in addition to *Sox9*, FGF2, Igf1, Tgfβ2 and Bmp2 enhance chondrocyte formation [14], [17]. Mutations in a number of genes including *Shh*, *Sox2*, retinoic acid synthesis genes and Fgf signaling pathway genes have been shown to affect cartilage ring formation [11]–[13], [18]–[21]. *Fgf10* mutants form a partial tracheal tube in spite of the failure of lung formation [8], [9]. Recent evidence shows that loss of *Fgf10* leads to defects in tracheal ring formation and that overexpression of *Fgf10* between E11.5 and E13.5 disrupts tracheal rings by altering the periodic expression of *Shh* in the trachea [22].

The T-box transcription factor genes are important during embryonic development. All members of this gene family contain a conserved DNA-binding T-box domain, which binds to a conserved sequence, the T-box binding element, to activate or repress transcription of specific target genes [23]. All Tbx2 subfamily genes, *Tbx2*, *Tbx3*, *Tbx4* and *Tbx5* are expressed in the developing chick lung buds and trachea between stages 15–21 [24]. In the mouse, *Tbx1* is expressed in lung epithelium at E12.5, *Tbx2* and *Tbx3* are expressed in lung mesenchyme at E11.5, and *Tbx4* and *Tbx5* are expressed in both lung and trachea mesenchyme at E12.5 and later [25]. *Tbx1* homozygous null mutants die at birth due to severe heart defects; the lungs are never fully inflated [26] but lung development has not been further investigated. In *Tbx4* homozygous mutants, lung buds form but the embryos die at E10.5 due to failure of allantois development and the subsequent lack of chorio-allantoic fusion leading to placental insufficiency [27]. *Tbx5* mutants die around E10 due to defects in heart development [28]; lung development has not been previously investigated. Antisense oligonucleotide depletion of both *Tbx4* and *Tbx5*, but not *Tbx2* and *Tbx3*, in lung organ cultures results in inhibition of branching and loss of *Fgf10* expression [29] suggesting a role for these factors in lung branching. In the chick embryo, interference with *Tbx4* function leads to a reduction in *Fgf10* expression in lung mesenchyme and inhibits lung bud formation. Ectopic expression of *Tbx4* leads to ectopic expression of *Fgf10* and *Nkx2.1* and lung bud formation in the esophagus. Additionally, ectopic expression of *Tbx4* at the boundary between the trachea and the esophagus can lead to lack of separation of these two structures, resulting in a tracheoesophageal fistula [30]. In humans, *Tbx5* mutations cause Holt Oram syndrome characterized by heart and forelimb abnormalities. A single de-novo mutation in *TBX5* has been linked to right lung agenesis [31].

To study the roles of *Tbx4* and *Tbx5* in lung and trachea development in the mouse, we made use of conditional alleles to bypass early embryonic lethality. We studied three distinct processes, namely 1) lung bud and trachea specification, 2) lung branching morphogenesis, and 3) tracheal/bronchial cartilage formation. We show that during early stages of development, *Tbx5* is important for specification of the lung buds and the trachea. After specification, *Tbx4* and *Tbx5* interact during lung growth and branching and the regulation of branching is dependent on *Fgf10* signaling. Additionally, *Tbx4* and *Tbx5* interact in the formation of mesenchymal condensations, which ultimately form the tracheal/bronchial cartilage rings independent of *Fgf10* signaling.

## Results

### Expression of *Tbx4* and *Tbx5* in the developing lung and trachea

**Figure 1:**
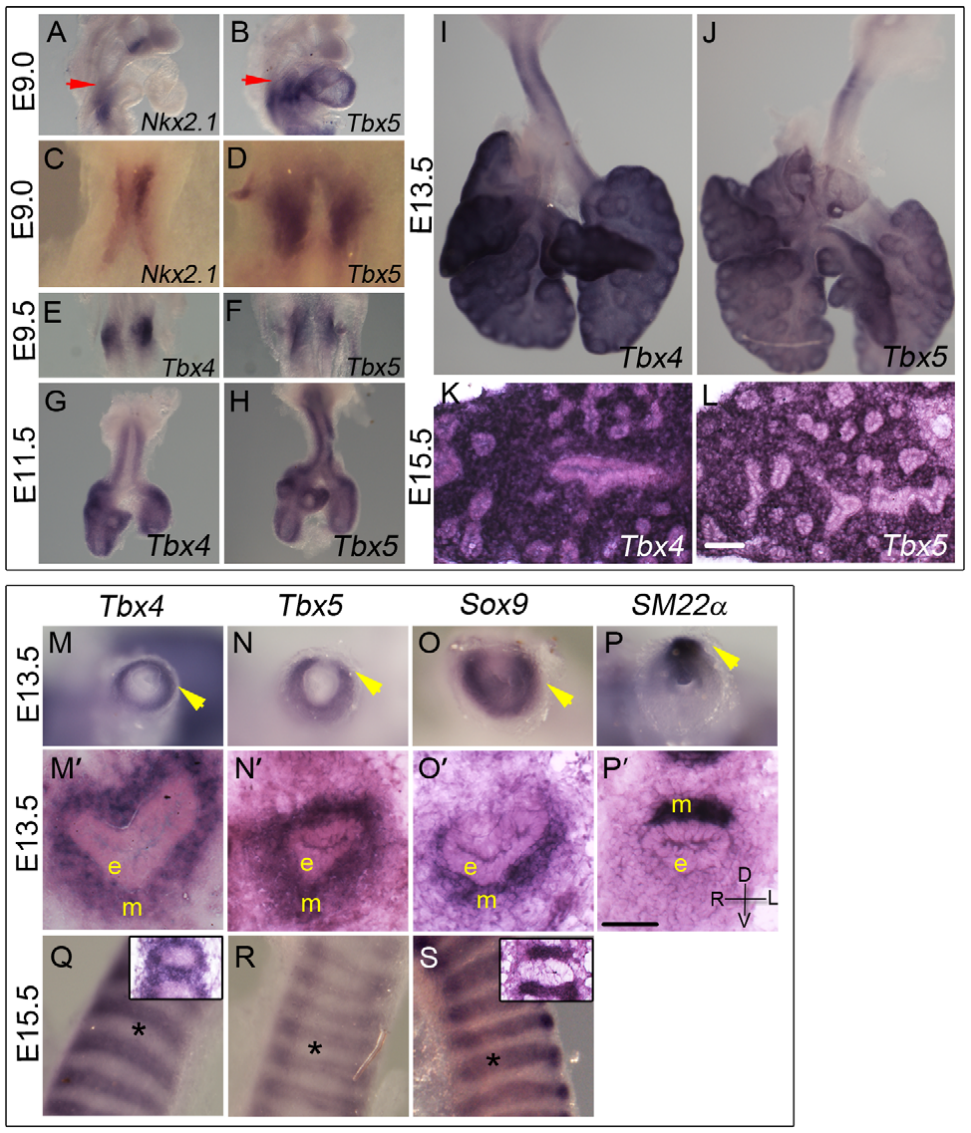
Expression of *Tbx4* and *Tbx5* in the developing lung and trachea. (A–L) *Tbx4* and *Tbx5* expression analyzed using ISH on lungs. *Tbx5* is first expressed at E9.0 (B,D) when the specification of lung primordia occurs, as seen by *Nkx2.1* expression (A,C). Red arrows point to the posterior extent of the third pharyngeal pouch which marks the anterior of the expression domain of both *Nkx2.1* and *Tbx5*. Right views (A,B), ventral views (C,D). *Tbx4* is first expressed at E9.5 along with *Tbx5* in the newly formed lung buds (E,F). Expression is seen in lung whole mounts at E11.5 (G,H), E13.5 (I,J) and in lung mesenchyme in cryosections at E15.5 (K,L). (M–S) *Tbx4* and *Tbx5* expression analyzed using ISH on tracheas. *Tbx4* and *Tbx5* are expressed throughout tracheal mesenchyme (m) at E13.5 (M,M′,N,N′) but not in the epithelium (e) or the mesothelium (arrowheads). Caudal view of cut tracheas after whole mount ISH (M–P). ISH on cryosections (M′–P′). At E13.5, *Sox9* is expressed in the mesenchyme on the ventral side (O,O′) and *SM22α* is expressed on the dorsal side (P,P′) of the trachea. D-dorsal; V-ventral; R-right; L-left. At E15.5, *Tbx4* and *Tbx5* are expressed around the condensing cartilage mesenchyme and in the intercartilage mesenchyme (Q,R). *Sox9* is expressed in the condensing cartilage rings (S). Asterisks indicate areas of cartilage condensation. Insets in (Q) and (S) show ISH on E15.5 sagittal cryosections with *Tbx4* and *Sox9* probe, respectively. Scale bars represent 50 µm.

*Tbx5* expression is first detected using *in situ* hybridization (ISH) at E9.0 (24 somites) in the mesenchyme of the lung and trachea primordia, concurrent with *Nkx2.1* expression in the ventral foregut epithelium (Figure 1A–1D). The anterior extent of expression of both genes coincides with the posterior extent of the third pharyngeal pouch (red arrow in Figure 1A, 1B). *Tbx4* expression is detected in the lung buds when they first appear a few hours later at E9.25 (28 somites) in a pattern similar to *Tbx5* (Figure 1E, 1F). *Tbx4* and *Tbx5* are expressed at E11.5, E13.5 and E15.5 throughout the lung mesenchyme but not in the epithelium (Figure 1G–1L) [25].

Both *Tbx4* and *Tbx5* show a dynamic expression pattern in the developing trachea. At E11.5 and E13.5 both genes are expressed throughout the tracheal mesenchyme surrounding the epithelium but are excluded from the epithelium and the outermost layer, the mesothelium (Figure 1G, 1H, 1M, 1M′, 1N, 1N′). At these stages, *Tbx4* and *Tbx5* are expressed in ventral tracheal cells that also express *Sox9* (Figure 1O, 1O′) [32] and in dorsal tracheal cells that also express *SM22α* (Figure 1P, 1P′) [33]. Within the ventral mesenchyme at E15.5, *Tbx4* and *Tbx5* expression is restricted to mesenchyme between and surrounding cartilage condensations (Figure 1Q, 1R) in a pattern complementary to that of *Sox9*, which is restricted to condensing mesenchyme of the cartilage rings at this stage (Figure 1S).

### Efficiency of recombination of conditional alleles

The genotypes of embryos used in this study and the corresponding descriptive shorthand nomenclature are shown in Table 1. PCR genotyping was used to determine the efficiency of recombination of the conditional alleles. For embryos carrying the tamoxifen-inducible *CreER* transgene, a dose of 8 mg tamoxifen was injected into pregnant females at E9.0 and embryos were dissected at E12.5. Yolk sacs were analyzed as an estimate of recombination in the whole embryo. Both alleles of *Tbx4^fl/fl^* embryos were completely recombined at E12.5 to produce the mutant allele at this dose of tamoxifen (Figure S1A), but the single floxed allele of *Tbx5^fl/+^* embryos was only partially recombined to the mutant form (Figure S1E). Doses of tamoxifen higher than 8 mg at E9.0 lead to a loss of pregnancy. When 7 mg tamoxifen was injected at E8.5, complete recombination of the *Tbx4^fl^* alleles (Figure S1B) and the single *Tbx5^fl^* allele (Figure S1F) was obtained at E13.5.

**Table 1:** Different allelic combinations and the descriptive nomenclature.

Genotype	Nomenclature
*Tbx4^fl/fl^;Tbx5^+/+^; CreER*	Conditional *Tbx4* null
*Tbx4^+/+^;Tbx5^fl/fl^; CreER*	Conditional *Tbx5* null
*Tbx4^fl/+^;Tbx5^fl/+^; CreER*	Conditional *Tbx4*;*Tbx5* double heterozygous
*Tbx4^fl/fl^;Tbx5^fl/+^; CreER*	Conditional *Tbx4* null;*Tbx5* heterozygous
*Tbx4^fl/fl^;Tbx5^fl/fl^; CreER*	Conditional double null
*Tbx4^cre/+^;Tbx5^fl/fl^*	Lung-specific *Tbx5* null
*Tbx4^cre/fl^;Tbx5^fl/fl^* 1 *Tbx4^cre/−^;Tbx5^fl/fl^* 1 *Tbx4^cre/fl^;Tbx5^fl/−^*	Lung-specific *Tbx4* heterozygous;*Tbx5* null

1 *Tbx4^−^* and *Tbx5^−^* alleles are generated by recombination of the floxed alleles in the germ line due to germ line expression of the *Tbx4^cre^* allele [38], [63].

In lung bud cultures a concentration of 1 µM 4-OH tamoxifen produced near-complete recombination of the *Tbx4^fl^* allele after 24 hours (Figure S1C) whereas the *Tbx5^fl^* allele was only partially recombined (Figure S1G). Virtually complete recombination of all floxed alleles was achieved after 4 days of culture (Figure S1D, S1H). These data suggest that the *Tbx5^fl^* allele has a lower efficiency of Cre-mediated recombination than the *Tbx4^fl^* allele. Thus we assume that there may be some residual Tbx5 activity from the *Tbx5^fl^* allele in the *in vivo* experiments, even in the presence of the *CreER* and *Tbx4^cre^* alleles.

### Early loss of *Tbx5*, but not *Tbx4*, leads to a unilateral loss of lung bud specification and absence of tracheal specification

**Figure 2:**
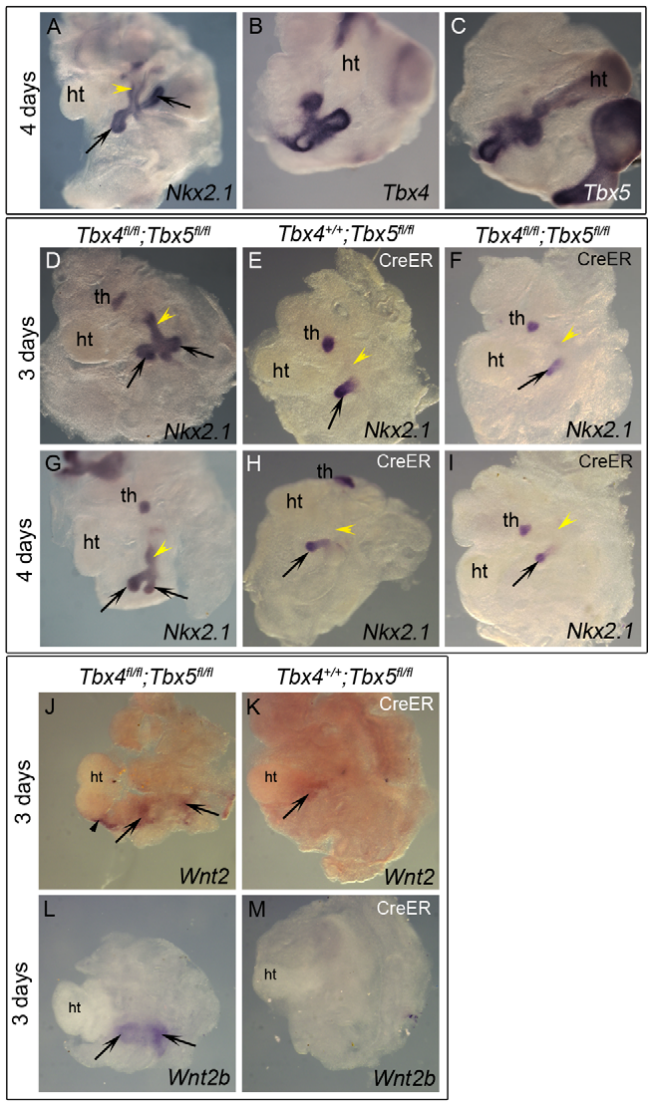
Early loss of *Tbx5* leads to aberrant lung bud and trachea specification. (A–I) Foreguts were isolated between 8–16 somite stages and analyzed by ISH following culture. *Nkx2.1* (A), *Tbx4* (B) and *Tbx5* (C) expression in lung buds (arrows) and tracheal primordia (yellow arrowhead) was confirmed in control cultures. Excision of *Tbx5* using 4-OH tamoxifen in conditional null foreguts with *CreER* leads to the loss of *Nkx2.1* expression in one of the lung buds at 3 (E) or 4 days (H). Conditional double nulls (F,I) carrying *CreER* show a phenotype similar to the conditional *Tbx5* nulls, suggesting additional removal of *Tbx4* does not exacerbate the phenotype. *Nkx2.1* expression was absent in the foregut tube of conditional *Tbx5* null and conditional double null foreguts after 3 or 4 days of culture (yellow arrowheads in E,F,H,I) compared to controls (D,G). Conditional *Tbx5* nulls show reduced *Wnt2* expression (K) and absence of *Wnt2b* expression (M) in the developing lung buds as compared to controls (J,L). Black arrowheads (J) point to *Wnt2* expression in the heart in the controls. ht, heart; th, thyroid primordia.

To explore the role of *Tbx4* and *Tbx5* in the earliest stages of lung and trachea specification, foregut culture [34] was used with *Nkx2.1* as a marker of specification. This *ex vivo* technique allows for analysis of mutants in culture, circumventing early embryonic lethality of the *Tbx4* homozygous mutants due to allantois defects and *Tbx5* homozygous mutants due to heart defects. When foreguts and surrounding tissue are isolated at E8.75 (8–16 somites), the foregut tube is devoid of *Nkx2.1* expression and the lung buds are not present [6]. At the end of 3 or 4 days of culture lung buds and trachea have formed as seen by *Nkx2.1* expression (Figure 2A, 2D, 2G). *Nkx2.1* is also expressed in the thyroid primordia at this stage (Figure 2D–2I and [35]). Expression of *Tbx4* and *Tbx5* was confirmed in control foreguts that were cultured for 4 days (Figure 2B, 2C). Foreguts from E8.5 embryos were cultured in the presence of 4-hydroxy (OH) tamoxifen and analyzed for *Nkx2.1* expression. Reduction of *Tbx5* alone lead to a lack of *Nkx2.1* expression in one of the lung buds after 3 or 4 days of culture (Figure 2E and 2H, respectively) suggesting a unilateral loss of lung bud specification. Removal of *Tbx4* alone did not affect *Nkx2.1* expression after 3 days of culture (data not shown) and removal of *Tbx4* in addition to *Tbx5* did not exacerbate the *Tbx5* phenotype (Figure 2F, 2I). Therefore, *Tbx5* but not *Tbx4* is important for the bilateral specification of lung buds *ex vivo*. *Wnt2* and *Wnt2b*, genes essential for specification of respiratory primordia [5], were analyzed in the conditional *Tbx5* null foreguts. *Wnt2* expression was reduced (Figure 2J, 2K) and *Wnt2b* expression was absent (Figure 2L, 2M) suggesting that *Tbx5* lies upstream of these genes in regulating the process of specification.

With respect to tracheal specification, *Nkx2.1* expression was not observed in the foregut tube after 3 or 4 days of culture in embryos lacking *Tbx5* (arrowheads in Figure 2E, 2H) suggesting a lack of tracheal specification in the absence of *Tbx5*. Additional loss of *Tbx4* alleles did not alter the phenotype (Figure 2F, 2I), supporting a role for *Tbx5* in the specification of the trachea, independent of *Tbx4*.

### Lung branching is severely affected by the loss of *Tbx4* and *Tbx5*

**Figure 3:**
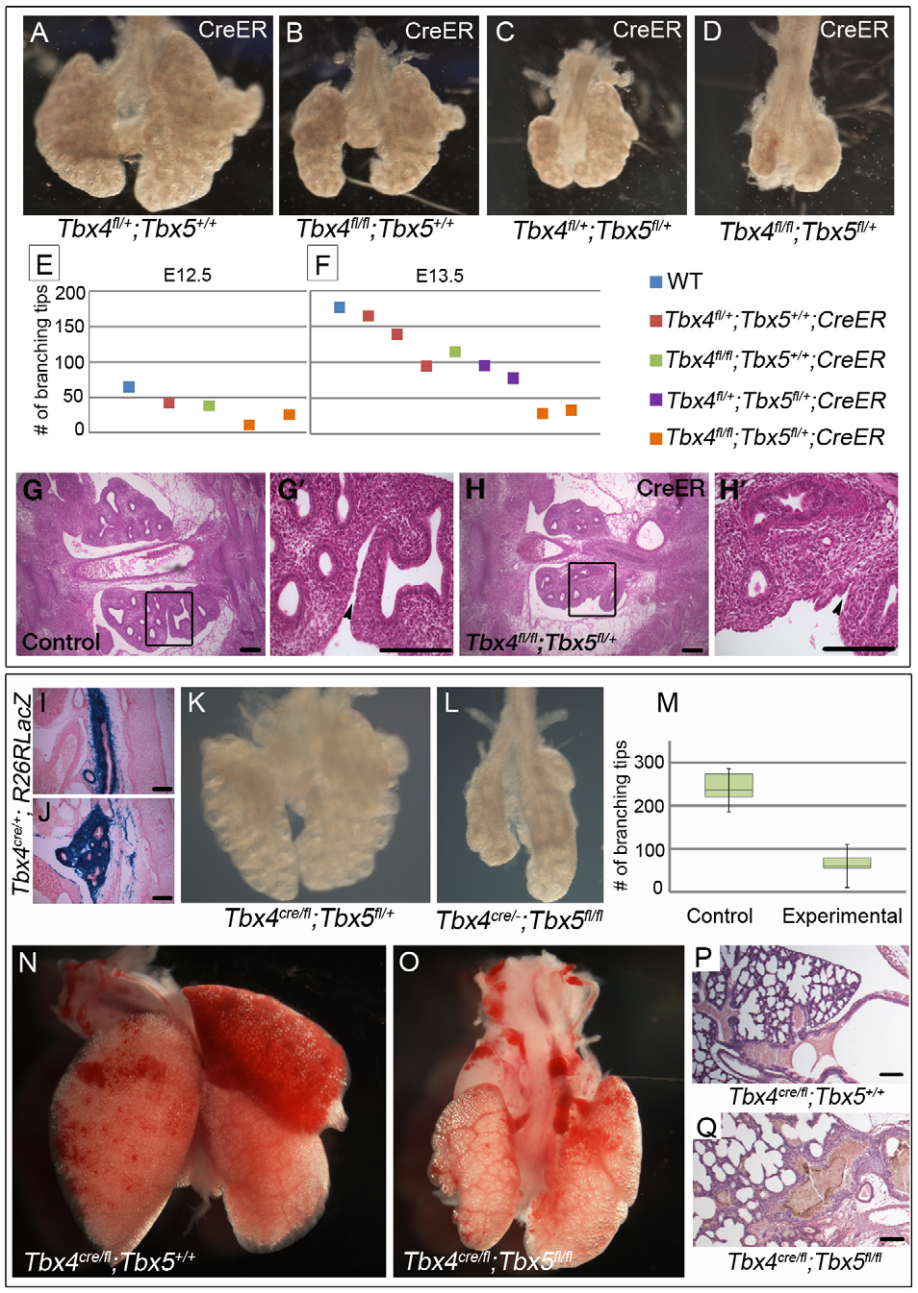
Loss of *Tbx4* and *Tbx5* causes reduced lung branching and lethality at birth. (A–H′) *Tbx4^fl^* and *Tbx5^fl^* alleles were excised using *CreER* by injecting tamoxifen at E8.75 and lungs were dissected at different time points. Loss of *Tbx4* alone or both *Tbx4* and *Tbx5* leads to lung hypoplasia at E13.5 (A–D). The number of branching tips was quantitated following E-cadherin staining for different genotypes at E12.5 (E) and E13.5 (F). H & E on histological sections shows a decrease in lung size at E12.5 in the conditional *Tbx4* null;*Tbx5* heterozygous lungs (G,H,). (G′) and (H′) are magnified views of the boxed regions in (G) and (H), respectively. Black arrowheads indicate separation between the lobes of the right lung in the control (G′) and lack of separation in the mutants (H′). (I–Q) *Tbx4^cre^* was utilized for lung and trachea-specific excision of *Tbx4^fl^* and *Tbx5^fl^* alleles. *Tbx4^cre^* was expressed in most of the trachea mesenchyme (I) and lung mesenchymal cells (J) as seen by a *R26RlacZ* reporter expression at E13.5. Lung-specific *Tbx4* heterozygous;*Tbx5* null lungs show hypoplasia at E13.5 (K,L) and at birth (N,O). The number of branching tips in control lungs and the lung-specific *Tbx4* heterozygous;*Tbx5* null lungs, labeled as experimental in the box plot (M), are significantly different at E13.5. The green boxes contain 50% of the values; the median is indicated by a horizontal line in the box; bars represent the 5^th^ and 95^th^ percentiles. H & E staining on sections shows lung morphology for control (P) and mutants (Q) at birth. Scale bars represent 100 µm.

To analyze the effect of loss of *Tbx4* and *Tbx5* on lung branching *in vivo* we made use of the tamoxifen-inducible *CreER* transgene. Tamoxifen was injected at E8.75 (8–16 somites), late enough to bypass lethality but well before lung branching begins. Embryos with *CreER* and different combinations of the *Tbx4^fl^* and *Tbx5^fl^* alleles were examined at E12.5 and E13.5. Conditional *Tbx4* null lungs were similar to controls at E12.5 but had fewer branching tips at E13.5 (Figure 3A, 3B, 3E, 3F). Conditional *Tbx4;Tbx5* double heterozygous lungs (Figure 3C) were smaller in size and had fewer branching tips than conditional *Tbx4* null lungs at E13.5 (Figure 3B) but were more advanced developmentally (Figure 3E, 3F) than conditional *Tbx4* null;*Tbx5* heterozygous lungs (Figure 3D), which were severely retarded. Lobation in the right lung was disrupted in conditional *Tbx4* null;*Tbx5* heterozygous lungs: the accessory lobe was missing and only rudimentary cranial and medial lobes were present (Figure 3H). The lobes had a fused appearance (Figure 3H′) suggesting a failure of separation. Histologically, there were no obvious structural defects other than an overall reduction in size (Figure 3G, 3G′, 3H, 3H′).

Conditional *Tbx4* null;*Tbx5* heterozygous mutants could not be analyzed later than E13.5, due to hematopoietic defects caused by Cre-induced apoptosis [36] and it was not possible to analyze conditional *Tbx5* null embryos using the inducible *CreER in vivo* due to lethal heart defects. To circumvent these limitations, we used the *Tbx4^cre^* allele, which is expressed in the lung and trachea but not in the heart [37]. From this allele, *Cre* is expressed in the majority of cells of the developing lung and trachea mesenchyme, as seen with lacZ reporter expression at E13.5 (Figure 3I, 3J) [38].

Lung-specific *Tbx5* null mutants, carrying a single copy of *Tbx4^cre^*, showed a range of phenotypes from an apparently normal lung to a severe decrease in lung size (data not shown). We hypothesize that the variability in phenotype is due to variable extent of recombination of the *Tbx5^fl^* allele (Figure S1). All lung-specific *Tbx4* heterozygous;*Tbx5* null pups (n = 13/13 from 5 litters) became cyanotic at birth and died shortly thereafter due to respiratory distress. Unlike the variable lung size in the lung-specific *Tbx5* null mutants, the lungs of these mutants were consistently smaller than controls at E13.5 (Figure 3K, 3L) and at birth (Figure 3N, 3O) and had significantly fewer branching tips at E13.5 (Figure 3M). At P0 histology of the lung-specific *Tbx4* heterozygous;*Tbx5* null lungs is comparable to controls although the mutant tracheas show accumulation of a mucus like substance (Figure 3P, 3Q). Epithelia of mutant lungs show expression of T1α and Pro-surfactant protein C (Pro-SPC) (Figure S2A–S2D), markers for alveolar cell differentiation [10], [19], suggesting that appropriate differentiation of the lung epithelium occurs in the lung-specific *Tbx4* heterozygous;*Tbx5* null lungs. These lungs show lobation defects very similar to the conditional *Tbx4* null;*Tbx5* heterozygous mutant lungs. The accessory lobe was absent in most embryos but when present showed less branching; the cranial and caudal lobes also showed decreased branching. The cranial, medial and caudal lobes were not separated (Figure S3A, S3B). In addition, although tertiary dorsal branches were present in these lungs, they were crowded together and the secondary lateral branches had outgrown a shorter distance compared to controls (Figure S3C, S3D)

**Figure 4:**
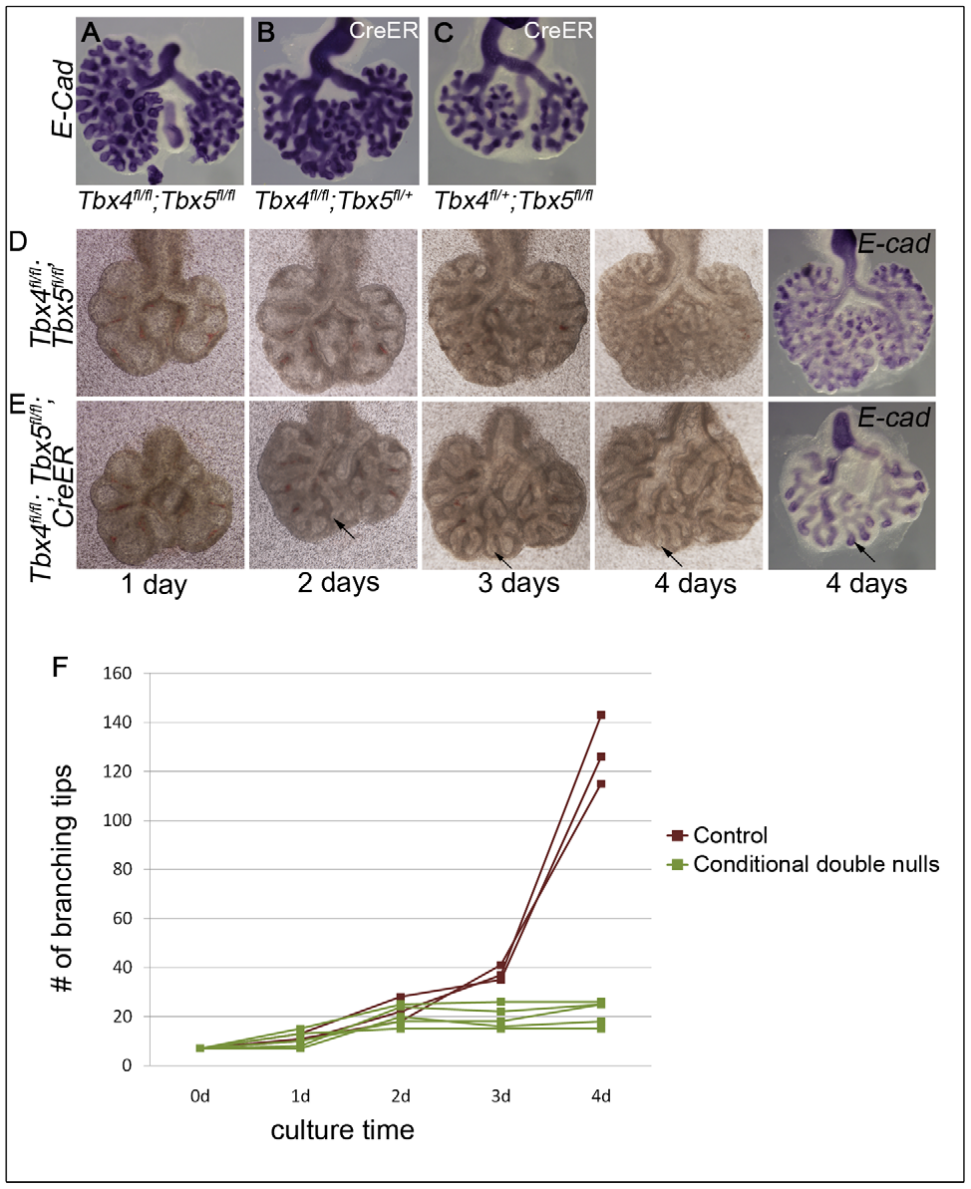
Loss of *Tbx4* and *Tbx5* leads to branching arrest *ex vivo*. Lung buds were isolated at E11.5 and cultured in the presence of 4-OH tamoxifen. At the end of the culture airways were visualized using *E-cadherin* ISH. Conditional *Tbx4* homozygous;*Tbx5* heterozygous (B) and conditional *Tbx4* heterozygous;*Tbx5* null (C) lungs with *CreER* showed reduced branching after 4 days of culture compared to controls (A). Control, without *CreER*, (D) and conditional double null lung buds, with *CreER*, (E) were cultured for 4 days and photographed at 1 day, 2 days, 3 days and 4 days. Arrows in E show the progression of an elongating airway. A plot of the number of branching tips as a function of time for controls and the conditional double nulls (F), shows a branching arrest of the conditional double null lungs after 2 days of culture (Mann Whitney U test p = 0.036).

Using *Tbx4^cre^,* it is not possible to study conditional double nulls as *Tbx4* is also expressed from this allele. Thus, to further explore branching morphogenesis in conditions where both alleles of *Tbx4* and *Tbx5* could be deleted, a lung bud culture system was used in which lung buds from embryos with or without the *CreER* transgene were cultured in the presence of 4-OH tamoxifen. Conditional *Tbx4* null;*Tbx5* heterozygous, or conditional *Tbx4* heterozygous;*Tbx5* null lung buds showed reduced branching (Figure 4A, 4B, 4C) consistent with the reduced number of branching tips observed *in vivo*. The conditional double null lungs showed a complete branching arrest by 3 days of culture (Figure 4D, 4E, 4F). The existing branches continued to elongate as seen at 4 days of culture (arrow in Figure 4E). Therefore, *Tbx4* and *Tbx5* are essential for continuing branching morphogenesis *ex vivo*.

### Loss of *Tbx4* and *Tbx5* affects the Fgf10 signaling pathway and *Wnt2* expression

**Figure 5:**
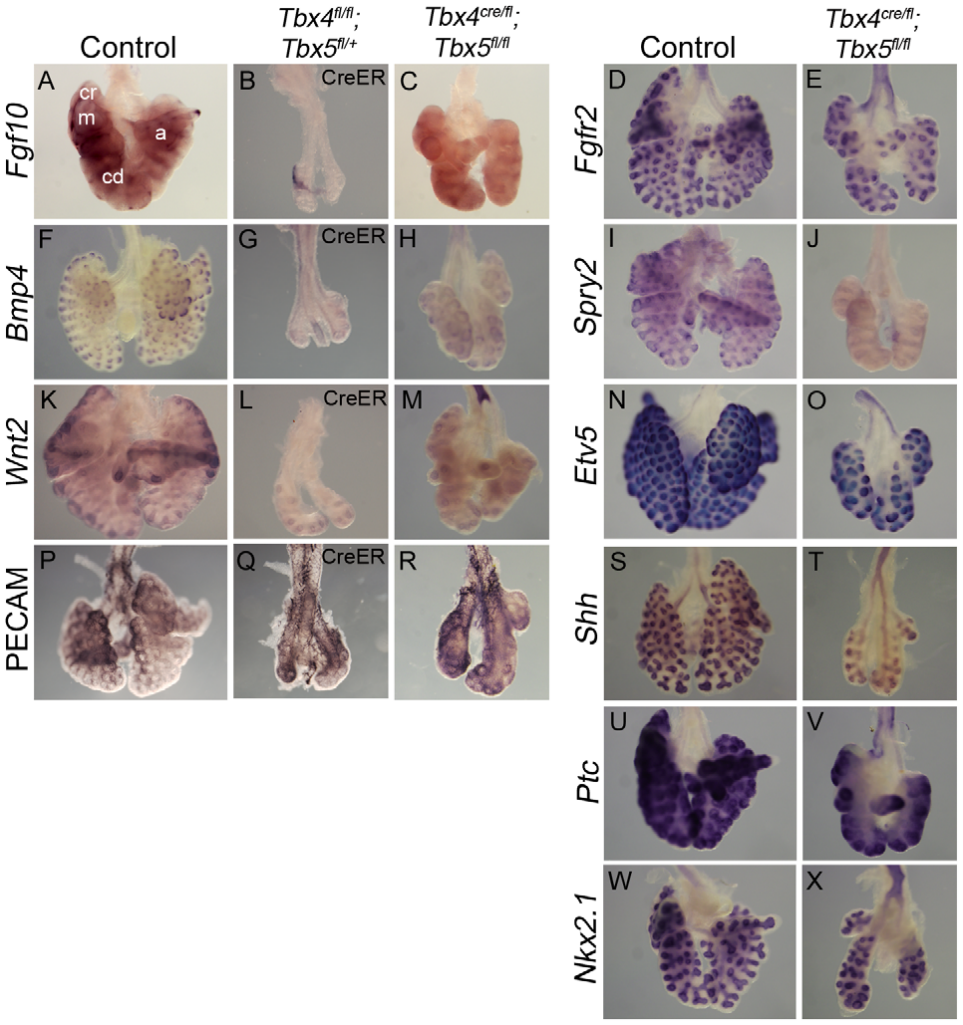
Loss of *Tbx4* and *Tbx5* affects the *Fgf10* signaling pathway and *Wnt2* expression. (A–X) Marker analysis of control and *Tbx4*- and *Tbx5*-deficient lungs using whole mount ISH (A–O, S–X) and IHC (P–R): Fewer foci of *Fgf10* expression were seen in the *Tbx4* and *Tbx5*-deficient lungs (B,C) compared to control (A). cr, cranial; m, medial; cd, caudal; a, accesory lobes. Fgf10 target genes *Bmp4* (F,G,H) and *Spry2* (I,J) and canonical *Wnt2* (K,L,M) were downregulated in *Tbx4* and *Tbx5*-deficient lungs compared to controls. *Fgfr2* (D,E), *Etv5* (N,O), PECAM (P,Q,R), *Shh* (S,T), *Ptc* (U,V) and *Nkx2.1* (W,X) were expressed similarly in controls and *Tbx4* and *Tbx5*-deficient lungs.

**Figure 6:**
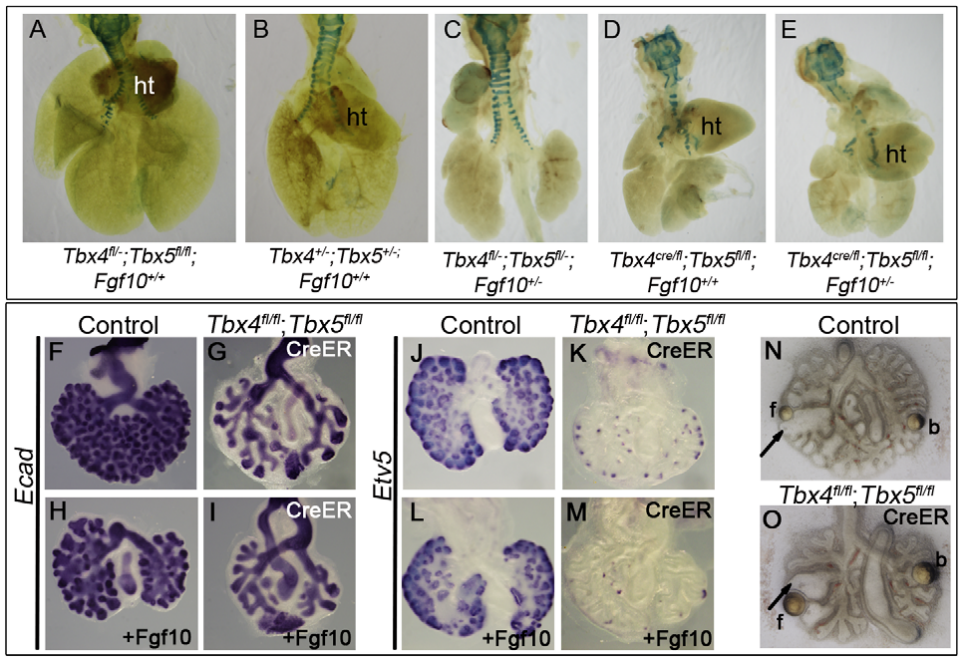
*Tbx4* and *Tbx5* interact with *Fgf10*, but FGF10 fails to rescue *Tbx4-* and *Tbx5*-deficient lungs. (A–E) Lungs of different genotypes at E18.5. *Tbx4*;*Tbx5* double heterozygous lungs (B) are smaller than control lungs (A). *Tbx4*;*Tbx5*;*Fgf10* triple heterozygous lungs (C) are smaller than the double heterozygous lungs (B) but comparable in size to the lung-specific *Tbx4* heterozyous;*Tbx5* null lungs (D). Removing an additional copy of Fgf10 from the lung-specific *Tbx4* heterozyous;*Tbx5* null lungs does not further affect lung size (E). ht, heart. (F–I) *E-cad* expression in epithelium of the control lungs and conditional double null lungs in the absence (F,G) and presence (H,I) of exogeneous Fgf10 showing a lack of rescue of branching morphogenesis. (J–M) *Etv5* expression in control lungs and conditional double null lungs in the absence (J,K) and presence (L,M) of exogeneous Fgf10. *Etv5* expression remains unchanged after addition of Fgf10. (N,O) Both control and conditional double null lungs respond to FGF10 coated beads (f) but not to BSA coated beads (b) as seen by swelling of tips (arrows) in proximity to the FGF10 bead.

Expression of the lung mesenchymal marker *Fgf10* as well as epithelial targets of the Fgf10 signaling pathway, *Bmp4*, *Spry2* and *Etv5* [39], [40], [41], was analyzed in lungs with reduced *Tbx4* and *Tbx5* expression. *Fgf10* is expressed in mesenchyme surrounding the distal epithelial tips that mark the site of future bud formation (Figure 5A) [42]. Consistent with the smaller overall lung size, there were fewer foci of *Fgf10* expression in the conditional *Tbx4* null;*Tbx5* heterozygous lungs (Figure 5B) and in the lung-specific *Tbx4* heterozygous;*Tbx5* nulls (Figure 5C). The primary receptor for this pathway, *Fgfr2* [43], is expressed normally in the epithelium of lung-specific *Tbx4* heterozygous;*Tbx5* null lungs (Figure 5D, 5E). *Bmp4* (Figure 5F, 5G, 5H) and *Spry2* (Figure 5I, 5J) were downregulated in *Tbx4* and *Tbx5-*deficient lungs but *Etv5* (Figure 5N, 5O) expression was not affected, although it was drastically reduced in conditional double nulls cultured *ex vivo* (see Figure 6J, 6K).

*Wnt2*, which is normally expressed in the developing lung mesenchyme (Figure 5K) [5], was greatly reduced at E13.5 in conditional *Tbx4* null;*Tbx5* heterozygous (Figure 5L) and lung-specific *Tbx4* heterozygous;*Tbx5* null lungs (Figure 5M). In addition to *Fgf10* and *Wnt2* signaling pathways, *Shh* signaling has also been implicated in branching morphogenesis in the lung [11]. Epithelial *Shh* (Figure 5S, 5T) and its mesenchymal receptor *Ptc* (Figure 5U, 5V) showed normal expression in lung-specific *Tbx4* heterozygous;*Tbx5* null lungs. The epithelial marker *Nkx2.1*, which is necessary for lung branching [44] and showed unilateral expression in the conditional *Tbx5* null foreguts at the time of specification (Figure 2), showed expression similar to controls at E13.5 in the lung-specific *Tbx4* heterozygous;*Tbx5* null lungs (Figure 5W, 5X). At E13.5, expression of the vascular marker Pecam indicated normal development of vessels around individual bronchioles of *Tbx4* and *Tbx5*-deficient mutants (Figure 5P, 5Q, 5R).

### *Tbx4* and *Tbx5* interact with *Fgf10* during lung development

Since mesenchymal *Fgf10* expression and expression of epithelial targets was affected in mutants with reduced *Tbx4* and *Tbx5*, we investigated the interactions between *Tbx4*, *Tbx5* and *Fgf10* during branching morhphogenesis using double and triple heterozygotus mutants. *Tbx4*;*Tbx5* double heterozygous lungs were smaller than control lungs at E18.5 (Figure 6A, 6B). *Tbx4*;*Tbx5*;*Fgf10* triple heterozygous lungs were smaller than *Tbx4*;*Tbx5* double heterozygous lungs (Figure 6B, 6C), suggesting that *Tbx4* and *Tbx5* genetically interact with the Fgf10 signaling pathway during lung development. Removing one copy of *Fgf10* from lung-specific *Tbx4* heterozygous;*Tbx5* nulls did not reduce lung size further (Figure 6D, 6E).

Despite the genetic interaction, the addition of exogenous Fgf10 to lung bud cultures of conditional double nulls did not rescue branching (Figure 6F–6I). The lack of change in *Etv5* expression, an *Fgf10* target, indicated that the Fgf10 signaling pathway was not activated in the presence of exogenous Fgf10 in the conditional double null lungs (Figure 6J–6M). To ensure that the Fgf10 used for the rescue experiments was active, Fgf10 coated heparin beads were placed near the branching tips of lung explants. The tips of both control and conditional double null lungs swelled up in response to Fgf10 beads but not BSA-coated heparin beads (Figure 6N, 6O) [45]. Since *Fgfr2* is expressed normally in the *Tbx4* and *Tbx5*-deficient lungs (Figure 5E) and the conditional double null lungs (data not shown), it is not surprising that the conditional double null lung tips can respond to Fgf10. However the conditional double null lungs fail to undergo branching in the presence of Fgf10. Thus, in addition to *Fgf10* there must be other factors under the control of *Tbx4* and *Tbx5* important for activation of the Fgf10 signaling pathway leading to branching morphogenesis.

### Loss of *Tbx4* and *Tbx5* causes tracheal/bronchial ring defects

**Figure 7:**
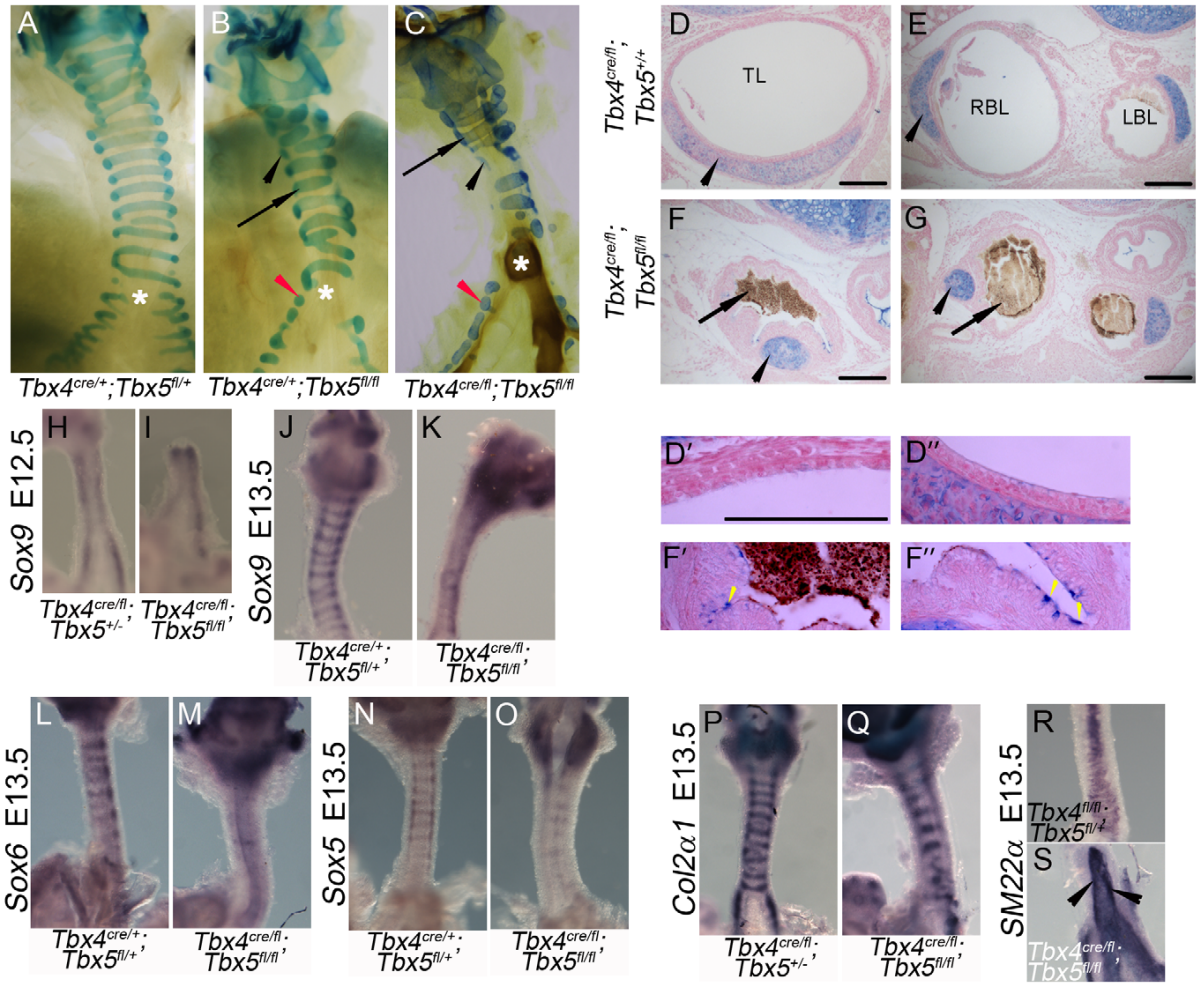
Loss of *Tbx4* and *Tbx5* disrupts tracheal/bronchial cartilage and smooth muscle formation. (A–G) Alcian blue staining was used to visualize tracheal/bronchial cartilage. Ten-eleven cartilage rings were seen in control tracheas (E18.5, A); normal (arrow) and incomplete rings were seen in tracheas and bronchi (black and red arrowheads respectively) of lung-specific *Tbx5* null (E18.5, B) and lung-specific *Tbx4* heterozygous;*Tbx5* nulls (postnatal day (P) 0, C). Asterisks in (A–C) indicate the point of bronchial separation from the trachea. Alcian blue on P0 sections showed that the control trachea (D) and main stem bronchial lumens (E) were expanded and the chondrocytes were present as C shaped rings (arrowheads). The lung-specific *Tbx4* heterozygous;*Tbx5* null tracheal lumen (F) and main stem bronchial lumen (G) were collapsed and filled with a mucus like substance (arrows) and alcian blue positive foci were seen (black arrowheads). Higher magnification views of control (D′,D″) and lung-specific *Tbx4* heterozygous;*Tbx5* null (F′,F″) tracheas. Yellow arrowheads indicate alcian blue positive mucus-producing cells in the lung-specific *Tbx4* heterozygous;*Tbx5* null tracheas. TL, Tracheal lumen; RBL, Right bronchial lumen; LBL, Left bronchial lumen. (H–S) Genes important for chondrogenesis and smooth muscle development. *Sox9* expression at E12.5 showed a comparable ventral expression pattern in control (H) and lung-specific *Tbx4* heterozygous;*Tbx5* null tracheas (I). At E13.5, *Sox9* positive cells begin to condense and appear in a ring like pattern in controls (J) but not in the mutant tracheas (K). *Sox6* (L,M) and *Sox5* (N,O) expression was downregulated in the lung-specific *Tbx4* heterozygous;*Tbx5* null tracheas at E13.5. *Col2α1* was expressed in its characteristic ring like pattern (P) similar to *Sox9* in the control tracheas but there were fewer rings with apparently normal expression in the lung-specific *Tbx4* heterozygous;*Tbx5* null tracheas (Q). *SM22α* expression was analyzed on the dorsal trachea at E13.5 in the control (R) and in the lung-specific *Tbx4* heterozygous;*Tbx5* null tracheas (S). Arrowheads in (S) point to ectopic expression in an uncharacteristic pattern in the mutants. Scale bars represent 100 µm.

To analyze the development of the tracheal/bronchial cartilage in embryos with reduced *Tbx4* and *Tbx5*, cartilage rings were visualized at birth using alcian blue staining. Lung-specific *Tbx5* nulls and lung-specific *Tbx4* heterozygous;*Tbx5* null embryos had defective cartilage ring development (Figure 7A–7C) with some normal rings (arrows in Figure 7B, 7C) and isolated foci of cartilage (black arrowheads in Figure 7B, 7C). The tracheal and bronchial lumen of newborn pups was expanded in the controls (Figure 7D, 7E) but collapsed in lung-specific *Tbx4* heterozygous;*Tbx5* nulls, and contained a mucus-like substance (Figure 7F, 7G). The tracheal epithelium of these mutants showed an increase in the number of mucus-producing cells, as seen by alcian blue staining (Figure 7D′, 7D″, 7F′, 7F″) [19]. To assess the development of cartilage rings at earlier stages, *Sox9* expression was analyzed at E12.5 and E13.5 in lung-specific *Tbx4* heterozygous;*Tbx5* nulls. At E12.5, these mutant tracheas have *Sox9* expression on the ventral aspect of the trachea similar to controls (Figure 7H, 7I) but fail to form mesenchymal condensations at E13.5 (Figure 7J, 7K). Expression of two genes genetically downstream of *Sox9, Sox6* (Figure 7L, 7M) and *Sox5* (Figure 7N, 7O), was downregulated at E13.5 whereas *Col2α1,* a *Sox9* target, was expressed at apparently normal levels in those rings that were present (Figure 7P, 7Q). The smooth muscle marker *SM22α* was analyzed to assess the development of the dorsal trachealis muscle. *SM22α* was expressed in an expanded domain and there was a loss of the characteristic banding pattern in the mutant tracheas (Figure 7R, 7S) indicating a disruption in smooth muscle formation due to loss of *Tbx4* and *Tbx5*.

### *Tbx4* and *Tbx5* do not interact with *Fgf10* during trachea development

**Figure 8:**
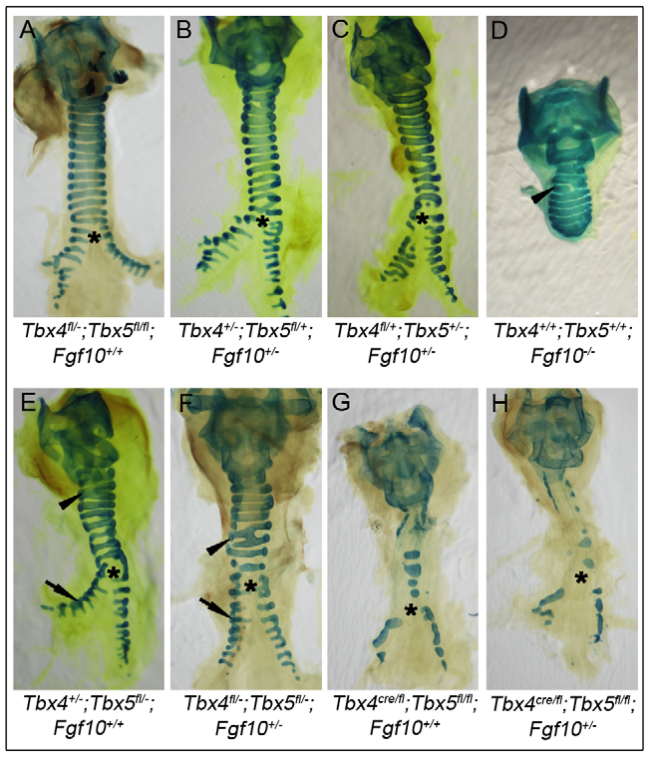
Lack of genetic interactions between *Tbx4*, *Tbx5*, and *Fgf10* in trachea formation. Alcian blue staining was used to analyze tracheal/bronchial cartilage development in tracheas from different genotypes at E18.5. Control tracheas (A), *Tbx4*;*Fgf10* double heterozygous tracheas (B) and *Tbx5*;*Fgf10* double heterozygous tracheas (C) have 10–11 C-shaped, ventral tracheal cartilage rings and the trachea forms the two main stem bronchi which have lateral C-shaped cartilage rings. *Fgf10* homozygous mutants (D), *Tbx4*;*Tbx5* double heterozygous mutants (E) and *Tbx4*;*Tbx5*;*Fgf10* triple heterozygous mutants (F) each form fewer (6–8) tracheal cartilage rings, some irregularly shaped or incomplete (arrowheads). Additionally, *Fgf10* mutants lack bronchi and hence any bronchial cartilage rings (D), but the double and triple heterozygotes form normal lateral bronchial cartilage rings (arrows in E and F). Lung-specific *Tbx4* heterozygous;*Tbx5* nulls (G) show severe disruptions in formation of tracheal/bronchial cartilage rings, a phenotype that is unchanged with the removal of an *Fgf10* allele (H). Asterisk shows the point of bronchial separation from the trachea.

Tracheas of controls (Figure 8A), *Tbx4*;*Fgf10* double heterozygotes (Figure 8B) and *Tbx5*;*Fgf10* double heterozygotes (Figure 8C) showed a normal pattern of 10–11 cartilage rings, suggesting a lack of genetic interactions between *Tbx4* or *Tbx5* and *Fgf10* in trachea formation. *Fgf10* null mutants do not form lungs but have a truncated trachea with 6–8 cartilage rings, some of which are aberrantly formed [22] (Figure 8D). *Tbx4*;*Tbx5* double heterozygous mice also have tracheas with 6–8 cartilage rings but in addition have main stem bronchial cartilage rings (Figure 8E). Removing a copy of *Fgf10* in these double heterozygotes did not alter the phenotype (Figure 8F) showing an *Fgf10*-independent role for *Tbx4* and *Tbx5* in the formation of tracheal/bronchial cartilage rings. Also, removing a copy of *Fgf10* in the lung-specific *Tbx4* heterozygous;*Tbx5* nulls did not alter the phenotype of the tracheal/bronchial cartilage rings (Figure 8G, 8H). Thus, *Tbx4* and *Tbx5* affect lung development via control of *Fgf10* expression but affect tracheal/bronchial cartilage development independently of the Fgf10 signaling pathway.

## Discussion

### Loss of *Tbx5* affects specification of the lung buds and trachea

**Figure 9:**
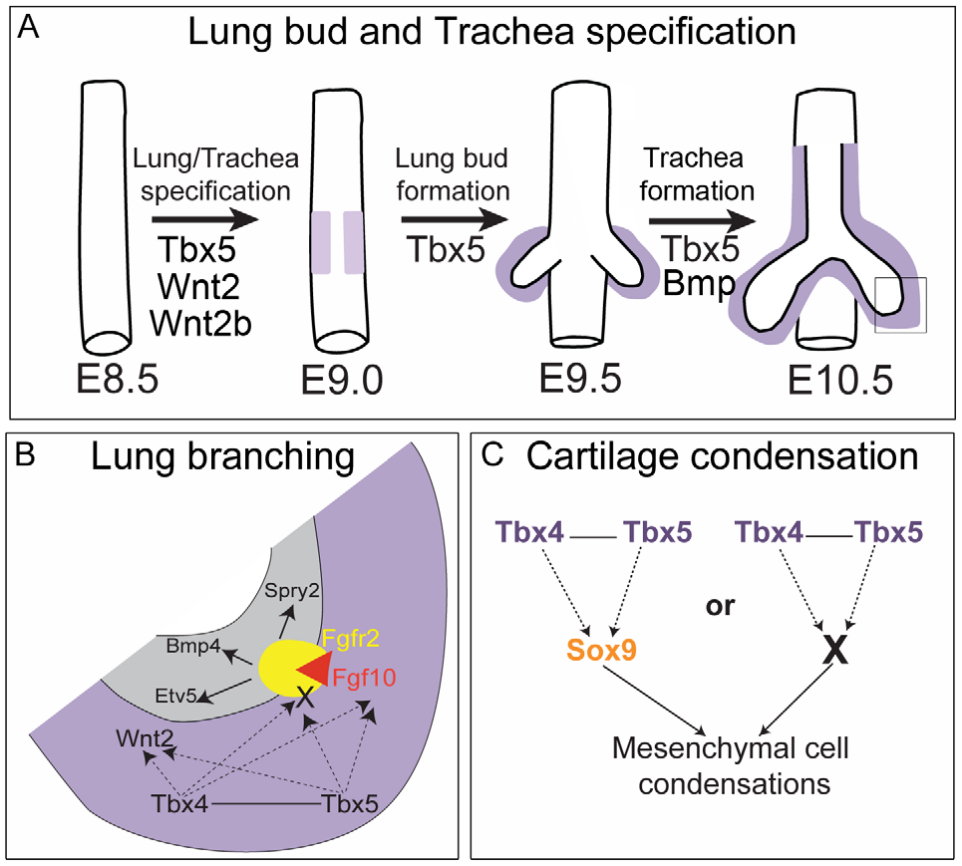
Model for the role of *Tbx4* and *Tbx5* in lung and trachea development. (A) Lung and trachea specification begins at E9.0 in the ventral foregut and at this time *Tbx5* expression (light purple) is adjacent to the presumptive endoderm. Later, *Tbx4* and *Tbx5* expression (dark purple) is in mesenchyme associated with the lung and trachea. *Tbx5* but not *Tbx4* is important for specification of bilateral lung buds and the trachea. (B) Magnification of box shown in (A) representing the events in the growing tip during branching morphogenesis. Grey denotes epithelium and purple denotes mesenchyme. *Tbx4* and *Tbx5* interact with each other and act upstream of the Fgf10 signaling pathway. Decrease in *Tbx4* and *Tbx5* affects mesenchymal *Fgf10* expression and expression of its targets in the epithelium – *Bmp4*, *Spry2* and *Etv5* – but not the expression of the epithelial Fgf10 receptor *Fgfr2*. In addition to *Fgf10* expression in the mesenchyme, *Tbx4* and *Tbx5* also control the expression of an unknown factor(s) (X) that is essential for activation of the Fgf10 signaling pathway. Furthermore, *Tbx4* and *Tbx5* act upstream of *Wnt2* in the mesenchyme. (C) In the trachea and the main stem bronchi *Tbx4* and *Tbx5* either control *Sox9* expression, which in turn regulates cartilage condensation, or *Tbx4* and *Tbx5* regulate another factor (X) essential for chondrogenesis secondarily affecting *Sox9* expression.

*Tbx5* is expressed around the lung/trachea primordia at the same time that *Nkx2.1*, a marker of specification, is first expressed in the primordia of the foregut endoderm. *Tbx4* is expressed slightly later at the time of lung bud formation. In our study, loss of *Tbx5*, but not *Tbx4*, leads to unilateral loss of lung bud specification, indicating that *Tbx5* has a distinct function in lung bud formation. In contrast, in the chick, although ectopic expression of *Tbx4* in the esophagus can specify lung fate, expression of a dominant negative form of *Tbx4* leads to a lack of primary budding in only a third of the mutants analyzed [30]. However, the dominant negative *Tbx4* could also be affecting the expression of *Tbx5* targets as the repressor construct utilized the complete T-box domain and *Tbx4* and *Tbx5* have 94% amino acid identity in their T-box domains [46]. This interpretation is compatible with our hypothesis that *Tbx5* plays a distinct role in vertebrate lung primordia specification (Figure 9A). Further our results suggest that *Tbx5* regulates specification by regulating the activity of *Wnt2* and *Wnt2b*.

Loss of *Tbx5* alone leads to a lack of tracheal specification *ex vivo*, a phenotype similar to that of mice mutant for *Bmpr1* and *Bmpr2*. In these mutants, although lung bud specification occurs, the ventral foregut fails to acquire tracheal identity [47]. Therefore, *Tbx5* either acts in parallel with or in the BMP signaling pathway in specification of the foregut into a trachea (Figure 9A).

### *Tbx4* and *Tbx5* interactions in lung development

In lung branching morphogenesis, *Tbx4* and *Tbx5* genetically interact with one another (Figure 9B). Although, neither *Tbx5* (data not shown) nor *Tbx4* single heterozygous lungs show a branching defect, double heterozygous lungs are smaller at E13.5 and E18.5 with a reduced number of branching tips at E13.5. Because these genes are closely related [46], they could potentially regulate the same target genes by binding to a similar T-box binding element, independently of one another. Alternatively, *Tbx4* and *Tbx5* could physically interact with each other as heterodimers to activate or repress transcription of downstream targets, as has been suggested for other T-box genes [48]. Even though Tbx4 and Tbx5 have a shared role in regulating lung branching, their relative contributions may not be equal, as *Tbx4*;*Tbx5* double heterozygous lungs are smaller than conditional *Tbx4* nulls. Unequal and distinct functions could be explained by domains outside of the T-box. For instance, the C terminal domain of both Tbx4 and Tbx5 has transcription activating capability which has been correlated to the shared limb outgrowth promoting activity of these genes [49] but Tbx4 also has a C terminal repressor domain which is proposed to be responsible for its distinct hind limb specific patterning activity [50]. In another example Tbx5 and Tbx4 both bind to a distinct LIM domain repeat of the LMP4 protein in the chick [51], illustrating distinct protein-protein interactions which could have functional implications.

In conditional *Tbx4* nulls;*Tbx5* heterozygous and lung-specific *Tbx4* heterozygous;*Tbx5* null lungs, reduction of *Tbx4* or *Tbx5* leads to a severe decrease in branching, a defect in formation of the lobes and failure of lobe separation, while absence of *Tbx4* and *Tbx5* leads to branching arrest. These results are in line with the published observations that antisense RNAs used against both *Tbx4* and *Tbx5* inhibit lung branching in culture and that this affect is explained by loss of Fgf10 expression [29]. Tbx5 binds and activates the *Fgf10* promoter *in vitro* suggesting that Fgf10 is a direct downstream target [52]. The drastic effect on lung branching in our study is explained by loss of expression of the important regulatory genes *Fgf10* and *Wnt2* in the lung mesenchyme. The Fgf10 targets *Bmp4* and *Spry2* show very low levels of expression in the epithelium of *Tbx4* and *Tbx5*-deficient mutants and *Etv5*, another Fgf10 target, shows greatly reduced expression in the conditional double null lungs, leading to the hypothesis that the *Fgf10* signaling pathway is activated downstream of *Tbx4* and *Tbx5* in the developing lung and that Fgf10 genetically interacts with *Tbx4* and *Tbx5*. Lungs that are triple heterozygous for *Tbx4*, *Tbx5* and *Fgf10* are reduced in size compared to *Tbx4*;*Tbx5* double heterozygous lungs, which supports this hypothesis. Lung-specific *Tbx4* heterozygote;*Tbx5* null lungs are severely retarded but lung size is not affected by further loss of *Fgf10*, demonstrating epistasis and supporting the hypothesis that *Fgf10* lies downstream of *Tbx4* and *Tbx5*. Lack of rescue of branch formation and lack of activation of the Fgf10 signaling pathway by exogenously supplied Fgf10 in culture suggests that there are other factors downstream of *Tbx4* and *Tbx5* that affect Fgf10 signaling. Although, lungs deficient in *Tbx4* and *Tbx5* retain the ability to respond to Fgf10 coated beads (our study and [29]), they fail to activate additional factors necessary for branching morphogenesis (X in Figure 9B).

One possibility for such a factor is a mesenchymal signaling molecule that communicates with the epithelium and activates downstream target(s) required for the activation of the Fgf10 pathway. We examined expression of *Ptc* and *Shh* to determine whether the Shh pathway was involved, but Shh signaling is not affected in the lung-specific *Tbx4* heterozygous;*Tbx5* nulls or in the conditional double null cultures (data not shown). The extracellular matrix (ECM) molecules, heparan sulfate (HS) proteoglycans aid in *Fgf10*-*Fgfr2* interactions during lung development [45] and hence are good candidates for factors missing in the *Tbx4* and *Tbx5*-deficient mutants. Inhibition of heparanase decreases submandibular gland morphogenesis in culture due to deficiency of Fgf signaling [53]. Additionally, HS-deficient null mouse embryos fail to respond to Fgf signaling and the spatiotemporal expression of cell surface-tethered HS chains regulates the local reception of Fgf-signaling activity during embryonic development [54]. *Tbx4* regulates ECM molecules in the developing allantois, specifically the chondroitin sulfate proteoglycan versican (R. Arora and V. E. Papaioannou unpublished observations), suggesting that ECM might be one of the targets for T-box genes in regulating development of other organs as well.

### *Tbx4* and *Tbx5* interactions in tracheal/bronchial cartilage development

Appropriate dorsal smooth muscle development and ventral tracheal/bronchial cartilage development is important for the normal functioning of the trachea. Smooth muscle provides tracheal flexibility and the cartilaginous rings prevent tracheal collapse. Defects in trachea formation may result in tracheomalacia or tracheal stenosis. Reduction of *Tbx4* and *Tbx5* causes defects in both formation of the tracheal cartilage rings and development of the trachealis smooth muscle indicating interactions between *Tbx4* and *Tbx5*. *Tbx4* and *Tbx5* heterozygous tracheas have 10–11 cartilage rings but *Tbx4;Tbx5* double heterozygous tracheas have 6–8 cartilage rings, some of which are incomplete. Additional reduction of *Tbx4* and *Tbx5* in the lung-specific *Tbx4* heterozygous;*Tbx5* null lungs leads to a complete disruption of cartilage ring formation supporting a genetic interaction between *Tbx4* and *Tbx5* in trachea formation.

*Sox9*, a master regulator of the process of chondrogenesis, is expressed normally at E12.5 throughout the ventral mesenchyme in lung-specific *Tbx4* heterozygous;*Tbx5* null tracheas but at E13.5 these tracheas show a lack of characteristic mesenchymal condensations and reduced *Sox9* expression. Either *Tbx4* and *Tbx5* control of *Sox9* expression becomes more sensitive to dosage at E13.5 or *Tbx4* and *Tbx5* control another factor, possibly an ECM molecule, which is important for formation of mesenchymal condensations and, in the absence of these condensations, there is a down regulation of *Sox9* expression (Figure 9C). Expression of *Sox5* and *Sox6*, genes genetically downstream of *Sox9* and important for condensation and cartilage formation, is downregulated in lung-specific *Tbx4* heterozygous;*Tbx5* null tracheas, concordant with an aberration in the process of chondrogenesis.

While *Tbx4* and *Tbx5* regulate lung branching by controlling Fgf10 signaling, their control of tracheal/bronchial cartilage formation is independent of Fgf10 signaling. *Fgf10* homozygous mutants have 6–8 tracheal cartilage rings, although the spacing between them is reduced and the rings do not always form the characteristic C shape [8], [9], [22]. Neither *Tbx4;Fgf10* double heterozygous tracheas nor *Tbx5*;*Fgf10* double heterozygous tracheas show cartilage condensation defects or a reduction in the number of cartilage rings. In contrast, *Tbx4*;*Tbx5* double heterozygotes show a shorter trachea with 6–8 tracheal cartilage rings suggesting *Tbx4* and *Tbx5* interact with each other during trachea formation but do not interact with *Fgf10*. The triple heterozygous tracheas do not show an exacerbation of the *Tbx4*;*Tbx5* double heterozygous trachea phenotype. Additionally, aberrant Fgf10 signaling has been shown to affect tracheal cartilage formation and loss of Fgf10 affects *Shh* expression but not *Sox9* expression [22]. In contrast, in the *Tbx4* and *Tbx5*-deficient mutants *Shh* expression appears to be unaffected whereas *Sox9* expression is reduced at E13.5. Hence, *Tbx4* and *Tbx5* control tracheal/bronchial cartilage formation via *Sox9*, independent of the Fgf10 signaling pathway.

## Materials and Methods

### Mouse strains, crosses, and embryo collection

Mice carrying the following alleles were genotyped as previously described: a Tbx4 conditional ‘floxed’ allele, *Tbx4^tm1.2Pa^* [55], hereafter referred to as *Tbx4^fl^*; a Tbx5 conditional floxed allele, *Tbx5^tm1.2Jse^* [56], hereafter referred to as *Tbx5^fl^*; an *Fgf10* null allele [8]; *ROSA26^CRE-ERT2^*, a ubiquitous tamoxifen-inducible *cre* transgene [57], hereafter referred to as *CreER*; *Tbx4-cre*, an insertion into the endogenous *Tbx4* allele resulting in a bicistronic allele that expresses both *cre* and *Tbx4* in all areas of *Tbx4* expression including lung and trachea [37], [38], hereafter referred to as *Tbx4^cre^*; and a *R26RlacZ* reporter [58]. All lines of mice were kept on mixed genetic backgrounds. Embryos were dissected from timed matings and yolk sacs were removed for PCR genotyping. The dark period was 19.00 to 05.00 h and noon on the day of a mating plug was identified as E0.5. All mouse work was carried out under Columbia University Medical Center Institutional Animal Care and Use Committee guidelines.

### Tamoxifen injections

Tamoxifen (Sigma) at a concentration of 20 mg/ml in sunflower oil (Sigma) was administered to pregnant females by intraperitoneal injection between 15.30 and 19.30 hours on E8.5 or between 23.00 and 24.00 hours on E9.0.

### *In situ* hybridization, immunohistochemistry, and histology

Whole-mount ISH, immunohistochemistry (IHC), immunofluorescence (IF) and ISH on cryosections was performed as described previously [59], [60], [61]. Primary antibodies used were anti-PECAM (Pharmingen, catalog number 01951D), anti-E-cadherin (Takara clone ECCD-2), anti T1α (Developmental Studies Hybridoma Bank antibody 8.1.1) and anti Prosurfactant Protein C (Millipore catalog number AB3786). All secondary antibodies were either peroxidase-conjugated donkey IgG from Jackson Immunochemicals or Alexa Fluor 488 from Invitrogen.

For histology embryos were removed from the uterine horns, dissected out of the decidua and fixed in Bouin's fixative (Sigma). After dehydration in ethanol, embryos were embedded in paraffin wax, sectioned at 8 µm thickness and stained with hematoxylin and eosin (H & E).

### Alcian blue staining

Alcian blue staining was performed according to standard protocols [62]. Lungs and trachea were dissected out at different stages, fixed in Bouin's fixative and washed with 70% ethanol. The tissue was then equilibrated in 5% acetic acid and stained with 0.05% alcian blue in 5% acetic acid for 2 hours. The tissue was washed in 5% acetic acid to remove excess stain and dehydrated in 100% methanol, cleared in BABB (benzyl alcohol, benzyl benzoate) and photographed. For alcian blue staining on sections, 8 µm paraffin sections were rehydrated, treated with 0.05% alcian blue in 5% acetic acid and then counterstained with nuclear fast red.

### Foregut and lung bud culture

Foregut culture was carried out as described previously [34]. Foreguts were isolated from 8–16 somite stage embryos using tungsten needles and cultured at 37°C in the presence of 95% air and 5% CO_2_ on Transwell-Col filters (Fisher Scientific) containing 1.5 ml BGJb media (Invitrogen) supplemented with 10% fetal bovine serum (FBS), 0.2 mg/ml vitamin C (Sigma) and 2 µM 4-OH tamoxifen (Sigma). For lung bud culture, lung buds were dissected at E11.5 in phosphate buffered saline (PBS) with 0.1% bovine serum albumin (Sigma) and cultured in media containing DMEM (Invitrogen) with 10% fetal bovine serum, 1% penicillin/streptomycin (Invitrogen) and 1 µM 4-OH tamoxifen on 3.0 µm filters (Millipore) or 0.4 µm Transwell filters (Fisher Scientific). Similar results were obtained using both types of filter; results reported are for experiments with Millipore filters. Where specified, Fgf10 (R&D) was added after 1 day of culture at a concentration of 500 ng/ml. In some experiments heparin beads coated with Fgf10 (100 µg/ml) or BSA (100 µg/ml) were placed near the branching tips of the explants after 1 day of culture. Transwell filters were used for the bead experiments.

### Lung branching analysis

Lungs were stained with either E-cadherin antibody using IHC or *E-cadherin* RNA probe using ISH. In case of whole mount lungs, lobes were separated and photographed to count the number of branching tips. The cultured lungs were photographed and the branching tips were counted. For some experiments, after *E-cadherin* ISH, lungs were post fixed in 4% PFA, washed with PBT, dehydrated in 100% methanol, cleared in BABB and then photographed.

## Supporting Information

Figure S1 Analysis of excision efficiency of *Tbx4^fl^* and *Tbx5^fl^*. Females carrying embryos with *Tbx4^fl^*, *Tbx5^fl^* and *CreER* alleles were injected with either 8 mg tamoxifen at E9.0 and dissected at E12.5 (A,E) or 7 mg tamoxifen at E8.5 and dissected at E13.5 (B,F). *Tbx4^fl/fl^* was completely excised to mutant *Tbx4^−/−^* at both doses and times (A,B) whereas the single conditional allele of *Tbx5^fl/+^* was incompletely excised by injection at E9.0 (E) but completely excised by injection at E8.5 (F). In addition when lungs with the genotype *Tbx4^fl/fl^*; *Tbx5^fl/fl^*; *CreER* were treated with 1 µm tamoxifen in culture, the *Tbx4* locus was nearly completely excised at the end of 1 day of culture (C) but the *Tbx5* locus was only partially excised (G). At the end of a 4 day culture both *Tbx4* (D) and *Tbx5* (H) loci achieved virtually complete excision. fl, floxed conditional PCR band; WT, wild type PCR band; Mut, excised PCR band.
(TIF)

Figure S2 Loss of *Tbx4* and *Tbx5* does not affect alveolar differentiation. (A,B) T1α IHC on cryosections of control and lung-specific *Tbx4* heterozygous;*Tbx5* null lungs at E18.5 shows comparable staining in the lung epithelium. Nuclear fast red was used as a counter stain. A′ and B′ are higher magnification views of boxed regions in A and B. (C,D) Prosurfactant protein C (Pro-SPC) IF on cryosections of control and lung-specific *Tbx4* heterozygous;*Tbx5* null lungs at E18.5 shows comparable staining in the lung epithelium. DAPI was used to stain the nuclei. Scale bars represent 100 µm.
(TIF)

Figure S3 Loss of *Tbx4* and *Tbx5* has multiple affects on branching morphogenesis. (A,B) H & E sections of lung-specific *Tbx4* heterozygous;*Tbx5* null lungs (B) show lack of separation of the cranial (cr), medial (m) and caudal (cd) lobes in the right lung as compared to control lungs (A). Black arrowheads point to the space created between the lobes in the control lungs (A) and to the corresponding regions of the mutant lungs (B). (C,D) Control lungs stained for *E-cadherin* at E13 show greater outgrowth (yellow parentheses) of lateral branches (C) than the lung-specific *Tbx4* heterozygous;*Tbx5* null lungs (D). Scale bars represent 100 µm.
(TIF)

## References

[1] Whitsett JA, Wert SE, Trapnell BC (2004). Genetic disorders influencing lung formation and function at birth.. Hum Mol Genet.

[2] Cardoso WV, Lu J (2006). Regulation of early lung morphogenesis: questions, facts and controversies.. Development.

[3] Spooner BS, Wessells NK (1970). Mammalian lung development: interactions in primordium formation and bronchial morphogenesis.. J Exp Zool.

[4] Metzger RJ, Klein OD, Martin GR, Krasnow MA (2008). The branching programme of mouse lung development.. Nature.

[5] Goss AM, Tian Y, Tsukiyama T, Cohen ED, Zhou D (2009). Wnt2/2b and [beta]-catenin signaling are necessary and sufficient to specify lung progenitors in the foregut.. Developmental Cell.

[6] Harris-Johnson KS, Domyan ET, Vezina CM, Sun X (2009). beta-catenin promotes respiratory progenitor identity in mouse foregut.. Proc Natl Acad Sci U S A.

[7] Domyan ET, Sun X (2011). Patterning and plasticity in development of the respiratory lineage.. Dev Dyn.

[8] Min H, Danilenko DM, Scully SA, Bolon B, Ring BD (1998). *Fgf-10* is required for both limb and lung development and exhibits striking functional similarity to Drosophila *branchless*.. Genes Dev.

[9] Sekine K, Ohuchi H, Fujiwara M, Yamasaki M, Yoshizawa T (1999). Fgf10 is essential for limb and lung formation.. Nat Genet.

[10] Weaver M, Yingling JM, Dunn NR, Bellusci S, Hogan BL (1999). Bmp signaling regulates proximal-distal differentiation of endoderm in mouse lung development.. Development.

[11] Pepicelli CV, Lewis PM, McMahon AP (1998). Sonic hedgehog regulates branching morphogenesis in the mammalian lung.. Curr Biol.

[12] Miller LA, Wert SE, Clark JC, Xu Y, Perl AK (2004). Role of *Sonic hedgehog* in patterning of tracheal-bronchial cartilage and the peripheral lung.. Dev Dyn.

[13] Mendelsohn C, Lohnes D, Decimo D, Lufkin T, LeMeur M (1994). Function of the retinoic acid receptors (RARs) during development (II) Multiple abnormalities at various stages of organogenesis in RAR double mutants.. Development.

[14] DeLise AM, Fischer L, Tuan RS (2000). Cellular interactions and signaling in cartilage development.. Osteoarthritis Cartilage.

[15] de Crombrugghe B, Lefebvre V, Nakashima K (2001). Regulatory mechanisms in the pathways of cartilage and bone formation.. Curr Opin Cell Biol.

[16] Akiyama H, Chaboissier MC, Martin JF, Schedl A, de Crombrugghe B (2002). The transcription factor Sox9 has essential roles in successive steps of the chondrocyte differentiation pathway and is required for expression of *Sox5* and *Sox6*.. Genes Dev.

[17] Hardingham TE, Oldershaw RA, Tew SR (2006). Cartilage, SOX9 and Notch signals in chondrogenesis.. J Anat.

[18] Park J, Zhang JJ, Moro A, Kushida M, Wegner M (2010). Regulation of *Sox9* by Sonic Hedgehog (*Shh*) is essential for patterning and formation of tracheal cartilage.. Dev Dyn.

[19] Que J, Luo X, Schwartz RJ, Hogan BL (2009). Multiple roles for Sox2 in the developing and adult mouse trachea.. Development.

[20] Vermot J, Niederreither K, Garnier JM, Chambon P, Dolle P (2003). Decreased embryonic retinoic acid synthesis results in a DiGeorge syndrome phenotype in newborn mice.. Proc Natl Acad Sci U S A.

[21] Tiozzo C, De Langhe S, Carraro G, Alam DA, Nagy A (2009). Fibroblast growth factor 10 plays a causative role in the tracheal cartilage defects in a mouse model of Apert syndrome.. Pediatr Res.

[22] Sala FG, Del Moral PM, Tiozzo C, Alam DA, Warburton D (2011). FGF10 controls the patterning of the tracheal cartilage rings via *Shh*.. Development.

[23] Naiche LA, Harrelson Z, Kelly RG, Papaioannou VE (2005). T-box genes in vertebrate development.. Annu Rev Genet.

[24] Gibson-Brown JJ, Agulnik SI, Silver LM, Papaioannou VE (1998). Expression of T-box genes *Tbx2*-*Tbx5* during chick organogenesis.. Mech Dev.

[25] Chapman DL, Garvey N, Hancock S, Alexiou M, Agulnik SI (1996). Expression of the T-box family genes, *Tbx1-Tbx5*, during early mouse development.. Dev Dyn.

[26] Jerome LA, Papaioannou VE (2001). DiGeorge syndrome phenotype in mice mutant for the T-box gene, *Tbx1*.. Nat Genet.

[27] Naiche LA, Papaioannou VE (2003). Loss of *Tbx4* blocks hindlimb development and affects vascularization and fusion of the allantois.. Development.

[28] Bruneau BG, Nemer G, Schmitt JP, Charron F, Robitaille L (2001). A murine model of Holt-Oram syndrome defines roles of the T-box transcription factor Tbx5 in cardiogenesis and disease.. Cell.

[29] Cebra-Thomas JA, Bromer J, Gardner R, Lam GK, Sheipe H (2003). T-box gene products are required for mesenchymal induction of epithelial branching in the embryonic mouse lung.. Dev Dyn.

[30] Sakiyama J, Yamagishi A, Kuroiwa A (2003). *Tbx4-Fgf10* system controls lung bud formation during chicken embryonic development.. Development.

[31] Tseng YR, Su YN, Lu FL, Jeng SF, Hsieh WS (2007). Holt-Oram syndrome with right lung agenesis caused by a de novo mutation in the *TBX5* gene.. Am J Med Genet A.

[32] Elluru RG, Whitsett JA (2004). Potential role of Sox9 in patterning tracheal cartilage ring formation in an embryonic mouse model.. Arch Otolaryngol Head Neck Surg.

[33] Badri KR, Zhou Y, Schuger L (2008). Embryological origin of airway smooth muscle.. Proc Am Thorac Soc.

[34] Chen F, Cao Y, Qian J, Shao F, Niederreither K (2010). A retinoic acid-dependent network in the foregut controls formation of the mouse lung primordium.. J Clin Invest.

[35] Desai TJ, Malpel S, Flentke GR, Smith SM, Cardoso WV (2004). Retinoic acid selectively regulates *Fgf10* expression and maintains cell identity in the prospective lung field of the developing foregut.. Dev Biol.

[36] Naiche LA, Papaioannou VE (2007). Cre activity causes widespread apoptosis and lethal anemia during embryonic development.. Genesis.

[37] Luria V, Krawchuk D, Jessell TM, Laufer E, Kania A (2008). Specification of motor axon trajectory by ephrin-B:EphB signaling: symmetrical control of axonal patterning in the developing limb.. Neuron.

[38] Naiche LA, Arora R, Kania A, Lewandoski M, Papaioannou VE (2011). Identity and fate of *Tbx4*-expressing cells reveal developmental cell fate decisions in the allantois, limb, and external genitalia.. Dev Dyn.

[39] Weaver M, Dunn NR, Hogan BL (2000). Bmp4 and Fgf10 play opposing roles during lung bud morphogenesis.. Development.

[40] Horowitz A, Simons M (2008). Branching morphogenesis.. Circ Res.

[41] Firnberg N, Neubuser A (2002). FGF signaling regulates expression of *Tbx2*, *Erm*, *Pea3*, and *Pax3* in the early nasal region.. Dev Biol.

[42] Bellusci S, Grindley J, Emoto H, Itoh N, Hogan BL (1997). Fibroblast growth factor 10 (FGF10) and branching morphogenesis in the embryonic mouse lung.. Development.

[43] Arman E, Haffner-Krausz R, Gorivodsky M, Lonai P (1999). *Fgfr2* is required for limb outgrowth and lung-branching morphogenesis.. Proc Natl Acad Sci U S A.

[44] Kimura S, Hara Y, Pineau T, Fernandez-Salguero P, Fox CH (1996). The T/ebp null mouse: thyroid-specific enhancer-binding protein is essential for the organogenesis of the thyroid, lung, ventral forebrain, and pituitary.. Genes Dev.

[45] Izvolsky KI, Shoykhet D, Yang Y, Yu Q, Nugent MA (2003). Heparan sulfate-FGF10 interactions during lung morphogenesis.. Dev Biol.

[46] Papaioannou VE, Goldin SN, Epstein CJ, Erickson RP, Wynshaw-Boris A (2008). Introduction to the T-box genes and their roles in develoopmental signaling pathways.. Inborn Errors of Development. The Molecular Basis of Clinical Disorders of Morphogenesis, 2^nd^ Edition.

[47] Domyan ET, Ferretti E, Throckmorton K, Mishina Y, Nicolis SK (2011). Signaling through BMP receptors promotes respiratory identity in the foregut via repression of *Sox2*.. Development.

[48] Goering LM, Hoshijima K, Hug B, Bisgrove B, Kispert A (2003). An interacting network of T-box genes directs gene expression and fate in the zebrafish mesoderm.. Proc Natl Acad Sci U S A.

[49] Duboc V, Logan MP (2011). Regulation of limb bud initiation and limb-type morphology.. Dev Dyn.

[50] Ouimette JF, Jolin ML, L'Honore A, Gifuni A, Drouin J (2010). Divergent transcriptional activities determine limb identity.. Nat Commun.

[51] Krause A, Zacharias W, Camarata T, Linkhart B, Law E (2004). Tbx5 and Tbx4 transcription factors interact with a new chicken PDZ-LIM protein in limb and heart development.. Dev Biol.

[52] Agarwal P, Wylie JN, Galceran J, Arkhitko O, Li C (2003). *Tbx5* is essential for forelimb bud initiation following patterning of the limb field in the mouse embryo.. Development.

[53] Patel VN, Knox SM, Likar KM, Lathrop CA, Hossain R (2007). Heparanase cleavage of perlecan heparan sulfate modulates FGF10 activity during ex vivo submandibular gland branching morphogenesis.. Development.

[54] Shimokawa K, Kimura-Yoshida C, Nagai N, Mukai K, Matsubara K (2011). Cell surface heparan sulfate chains regulate local reception of FGF signaling in the mouse embryo.. Dev Cell.

[55] Naiche LA, Papaioannou VE (2007). *Tbx4* is not required for hindlimb identity or post-bud hindlimb outgrowth.. Development.

[56] Mori AD, Zhu Y, Vahora I, Nieman B, Koshiba-Takeuchi K (2006). Tbx5-dependent rheostatic control of cardiac gene expression and morphogenesis.. Dev Biol.

[57] de Luca C, Kowalski TJ, Zhang Y, Elmquist JK, Lee C (2005). Complete rescue of obesity, diabetes, and infertility in *db/db* mice by neuron-specific LEPR-B transgenes.. J Clin Invest.

[58] Soriano P (1999). Generalized *lacZ* expression with the ROSA26 Cre reporter strain.. Nat Genet.

[59] Wilkinson DG, Nieto MA (1993). Detection of messenger RNA by in situ hybridization to tissue sections and whole mounts.. Methods Enzymol.

[60] Davis CA (1993). Whole-mount immunohistochemistry.. Methods Enzymol.

[61] Grieshammer U, Le M, Plump AS, Wang F, Tessier-Lavigne M (2004). SLIT2-mediated ROBO2 signaling restricts kidney induction to a single site.. Dev Cell.

[62] Nagy A, Gertsenstein M, Vintersten K, Behringer R (2009). Alcian blue staining of the mouse fetal cartilaginous skeleton.. Cold Spring Harbor Protocols.

[63] Douglas NC, Heng K, Sauer MV, Papaioannou VE (2011). Dynamic expression of Tbx2 subfamily genes in development of the mouse reproductive system.. Dev Dyn.

