# Supplementary figures and images for: Multiple Roles and Interactions of Tbx4 and Tbx5 in Development of the Respiratory System
Source: PLoS Genet. 2012 Aug 2;8(8):e1002866. doi: 10.1371/journal.pgen.1002866 (PMC3410851; doi:10.1371/journal.pgen.1002866)

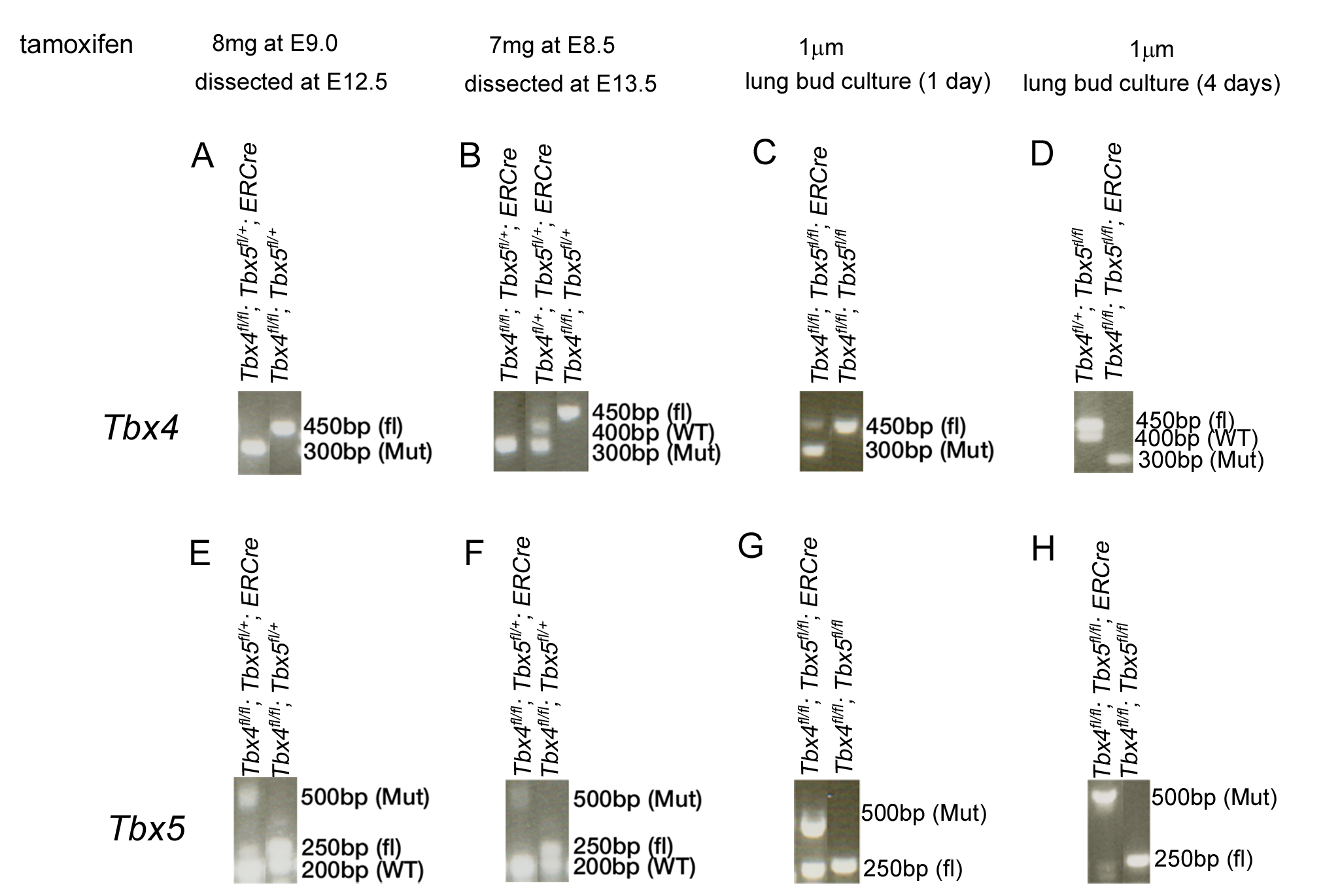

Supplement: Figure S1 — Analysis of excision efficiency of Tbx4fl and Tbx5fl. Females carrying embryos with Tbx4fl, Tbx5fl and CreER alleles were injected with either 8 mg tamoxifen at E9.0 and dissected at E12.5 (A,E) or 7 mg tamoxifen at E8.5 and dissected at E13.5 (B,F). Tbx4fl/fl was completely excised to mutant Tbx4−/− at both doses and times (A,B) whereas the single conditional allele of Tbx5fl/+ was incompletely excised by injection at E9.0 (E) but completely excised by injection at E8.5 (F). In addition when lungs with the genotype Tbx4fl/fl; Tbx5fl/fl; CreER were treated with 1 µm tamoxifen in culture, the Tbx4 locus was nearly completely excised at the end of 1 day of culture (C) but the Tbx5 locus was only partially excised (G). At the end of a 4 day culture both Tbx4 (D) and Tbx5 (H) loci achieved virtually complete excision. fl, floxed conditional PCR band; WT, wild type PCR band; Mut, excised PCR band. (TIF) [file pgen.1002866.s001.tif]

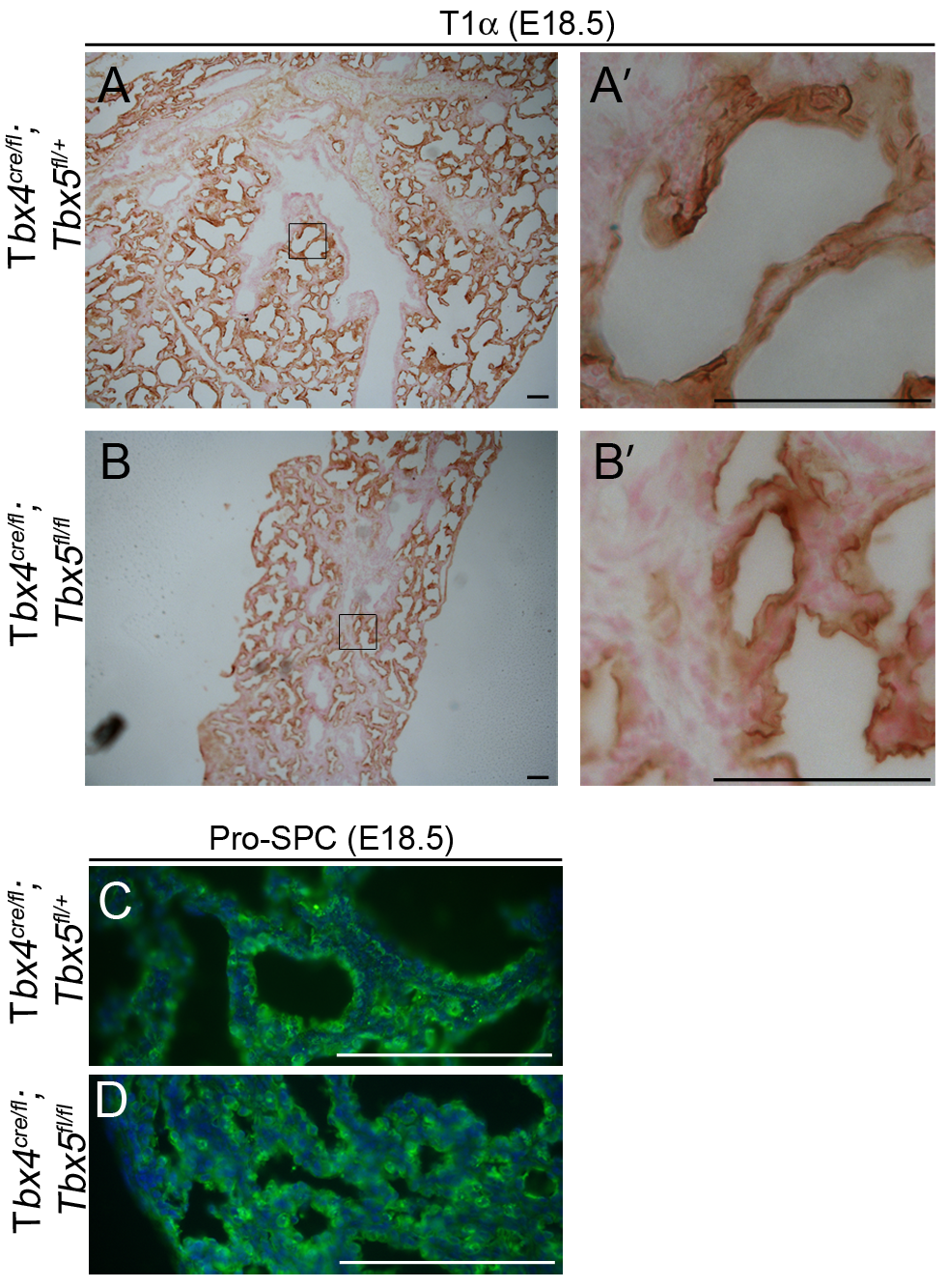

Supplement: Figure S2 — Loss of Tbx4 and Tbx5 does not affect alveolar differentiation. (A,B) T1α IHC on cryosections of control and lung-specific Tbx4 heterozygous;Tbx5 null lungs at E18.5 shows comparable staining in the lung epithelium. Nuclear fast red was used as a counter stain. A′ and B′ are higher magnification views of boxed regions in A and B. (C,D) Prosurfactant protein C (Pro-SPC) IF on cryosections of control and lung-specific Tbx4 heterozygous;Tbx5 null lungs at E18.5 shows comparable staining in the lung epithelium. DAPI was used to stain the nuclei. Scale bars represent 100 µm. (TIF) [file pgen.1002866.s002.tif]

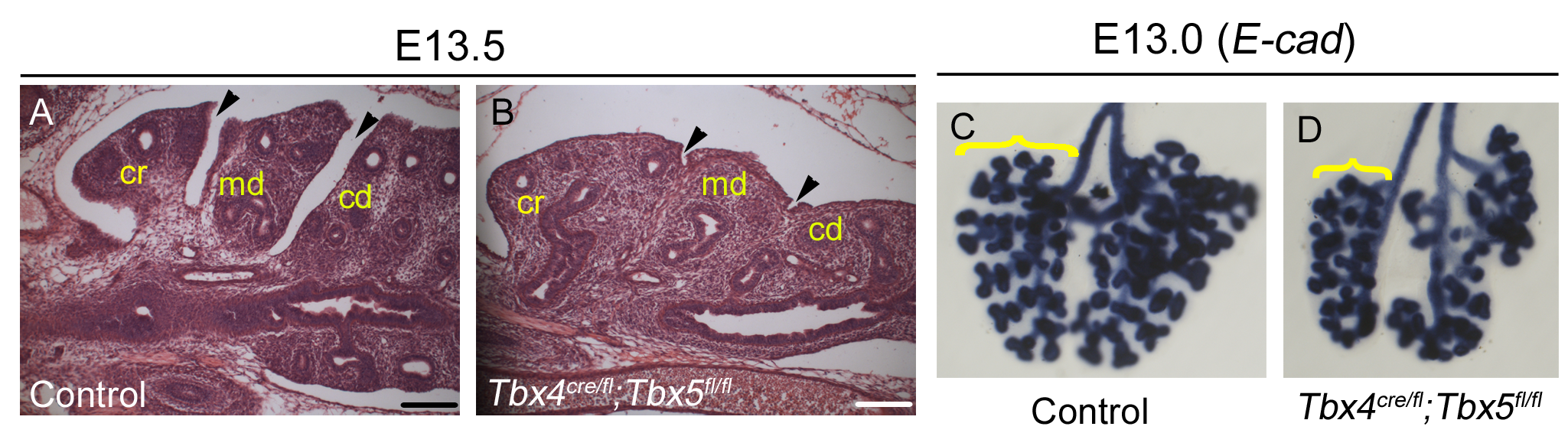

Supplement: Figure S3 — Loss of Tbx4 and Tbx5 has multiple affects on branching morphogenesis. (A,B) H & E sections of lung-specific Tbx4 heterozygous;Tbx5 null lungs (B) show lack of separation of the cranial (cr), medial (m) and caudal (cd) lobes in the right lung as compared to control lungs (A). Black arrowheads point to the space created between the lobes in the control lungs (A) and to the corresponding regions of the mutant lungs (B). (C,D) Control lungs stained for E-cadherin at E13 show greater outgrowth (yellow parentheses) of lateral branches (C) than the lung-specific Tbx4 heterozygous;Tbx5 null lungs (D). Scale bars represent 100 µm. (TIF) [file pgen.1002866.s003.tif]
